# Radiosynthesis of the anticancer nucleoside analogue Trifluridine using an automated ^18^F-trifluoromethylation procedure†
†Electronic supplementary information (ESI) available: ^1^H NMR and ^13^C NMR data for characterised compounds, automation setup, HPLC traces from radiosynthesis, Log *D*_7.4_ and radiotracer stability procedures, raw data and HPLC traces from all *in vitro* and *in vivo* studies. See DOI: 10.1039/c8ob00432c


**DOI:** 10.1039/c8ob00432c

**Published:** 2018-04-09

**Authors:** Alice King, Andreas Doepner, David Turton, Daniela M. Ciobota, Chiara Da Pieve, Anne-Christine Wong Te Fong, Gabriela Kramer-Marek, Yuen-Li Chung, Graham Smith

**Affiliations:** a Department of Radiotherapy and Imaging , Institute of Cancer Research , 123 Old Brompton Road , London , SW7 3RP , UK . Email: graham.smith@icr.ac.uk ; Tel: +44 (0)20 8722 4482

## Abstract

Trifluoromethyl groups are widespread in medicinal chemistry, yet there are limited ^18^F-radiochemistry techniques available for the production of the complementary PET agents. Herein, we report the first radiosynthesis of the anticancer nucleoside analogue trifluridine, using a fully automated, clinically-applicable ^18^F-trifluoromethylation procedure. [^18^F]Trifluridine was obtained after two synthetic steps in <2 hours. The isolated radiochemical yield was 3% ± 0.44 (*n* = 5), with a radiochemical purity >99%, and a molar activity of 0.4 GBq μmol^–1^ ± 0.05. Biodistribution and PET-imaging data using HCT116 tumour-bearing mice showed a 2.5 %ID g^–1^ tumour uptake of [^18^F]trifluridine at 60 minutes post-injection, with bone uptake becoming a prominent feature thereafter. *In vivo* metabolite analysis of selected tissues revealed the presence of the original radiolabelled nucleoside analogue, together with deglycosylated and phosphorylated [^18^F]trifluridine as the main metabolites. Our findings suggest a potential role for [^18^F]trifluridine as a PET radiotracer for elucidation of drug mechanism of action.

## Abstract 2

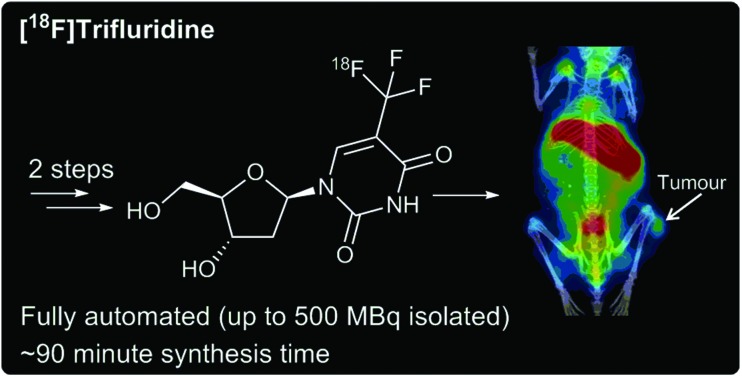
Automated ^18^F-trifluoromethylation provided the first radiosynthesis of [^18^F]Trifluridine, which shows potential as a PET probe of drug mechanism of action.

## Introduction

For many years, medicinal chemists have exploited the properties of the fluorine atom (particularly its small size, high electronegativity, high lipophilicity, and its strong bond with carbon) to induce favourable drug pharmacology.^1^,^2^ The direct incorporation of a single fluorine atom onto an aliphatic or aromatic carbon atom remains a challenge, due to the high reactivity and poor selectivity associated with electrophilic sources of fluorine, and the poor nucleophilicity of fluoride-derived reagents. In comparison, trifluoromethylation has shown improved substrate tolerance and reactivity. The availability of electrophilic,^3^,^4^ radical,^5^ and nucleophilic^6^ reagents provides a diverse chemical toolkit, enabling the trifluoromethylation of substrates with differing electronic configurations. The availability of operative synthetic procedures, coupled with the desired pharmacological effects that can be induced by the inclusion of three fluorine atoms, has meant that the trifluoromethyl group is now an increasingly common feature of pharmaceutical agents.^7^–^9^

The increasing presence of fluorine in drug molecules has created the opportunity to probe biological behaviour using positron emission tomography (PET). PET is a highly-sensitive, non-invasive imaging technique that is widely used for the clinical assessment of disease state, but has also proven highly valuable for applications in drug discovery^10^,^11^ and drug delivery.^12^ The high sensitivity of PET enables data acquisition through the administration of very small masses of compound, termed “microdosing”, which provides an associated safety benefit.^13^ Fluorine-18 (^18^F) is sometimes considered the optimum PET radioisotope, due to its moderate half-life (109.8 minutes) and the medium positron energy (≤0.635 MeV) that allows acquisition of high resolution images. Drug substances that are radiolabelled with fluorine-18 can be monitored using PET in an *in vivo* context, providing real-time pharmacokinetic data and enabling predictions of treatment response, metabolic stability, and effective dosing regimens. Despite the clear benefits associated with radiolabelling drug compounds, and the abundance of the trifluoromethyl group in such molecules, few ^18^F-trifluoromethylated PET probes have been reported in the literature.^14^–^17^

Historically, small molecules have been ^18^F-trifluoromethylated *via*^19^F/^18^F isotopic exchange^18^,^19^ or through nucleophilic displacement of a difluoromethyl halide.^15^,^20^,^21^ However, the widespread application of these methods has been limited for reasons including harsh reaction conditions, low molar activity, and challenging precursor synthesis. In recent years, there has been a surge in the number of reported ^18^F-trifluoromethylation procedures, with available methods now including copper-catalysed ^18^F-trifluoromethylation of aryl halides,^22^,^23^ aryl boronic acids,^24^,^25^ and benzaldehydes,^26^ as well as benzylic CH activation/^18^F-fluorination of difluoromethylarenes.^27^ Although these protocols continue to suffer some limitations (including generally low molar activities), these advances provide greater scope for the ^18^F-trifluoromethylation of structurally complex drug targets.^28^

5-Trifluoromethyl-2′-deoxyuridine, or trifluridine (TFT), is the active component of the combination chemotherapeutic agent TAS-102, which gained FDA approval for the treatment of metastatic colorectal cancer in 2015. Elucidating the mode of action and the pathways associated with TFT metabolism is a growing area of research,^29^,^30^ therefore a PET imaging probe may provide a useful tool for the investigation of *in vivo* response to TFT. As TFT is a substrate for the DNA salvage pathway enzyme thymidine kinase 1 (TK1),^31^ a [^18^F]TFT PET probe may also serve as a proliferation imaging agent, in a similar manner to 3′-deoxy-3′-[^18^F]fluorothymidine ([^18^F]FLT).^32^ The phosphorylation of [^18^F]FLT by TK1 leads to an accumulation of the imaging agent within proliferating cells. However, the ^18^F-fluorine 3′-substituent prevents sugar-phosphate backbone formation and subsequent DNA incorporation, which can result in radiotracer degradation through the action of a putative nucleotidase.^33^ Unlike [^18^F]FLT, TFT is incorporated into DNA,^31^ which may be advantageous in terms of radiotracer uptake and retention.

The aims of our study were two-fold: firstly, to provide the first radiosynthesis of [^18^F]TFT, using a broadly applicable, automated ^18^F-trifluoromethylation procedure suitable for clinical translation; and secondly, to assess the preliminary biological characteristics of [^18^F]TFT, as a potential PET imaging agent for evaluation of drug action and proliferation.

## Results and discussion

### Chemistry

The most efficient synthetic routes towards non-radioactive TFT involve the direct trifluoromethylation of 2′-deoxyuridine, using reagents that generate radical intermediates, such as the Togni^34^ and Langlois^35^ reagents. As yet, there are no radiolabelled derivatives of such reagents for ^18^F-trifluoromethylation. To design a suitable precursor for radiosynthesis, we evaluated several of the previously reported ^18^F-trifluoromethylation procedures. As we aimed to develop an automated and clinically translatable radiosynthesis of [^18^F]TFT, we required an operationally simple procedure that used a nucleophilic source of fluorine-18, as well as stable, easy-to-handle precursors and reagents. We therefore elected not to investigate methods involving the generation of gaseous [^18^F]HCF_3_,^24^,^25^ nor did we consider the benzylic CH activation methodology, which required the portionwise solid addition of iodosobenzene throughout the reaction.^27^ Instead, we pursued the copper-catalysed ^18^F-trifluoromethylation of aryl iodides reported by Huiban *et al.*,^22^ the effectiveness of which has been demonstrated on a structurally-related uracil precursor.

**Scheme 1:**
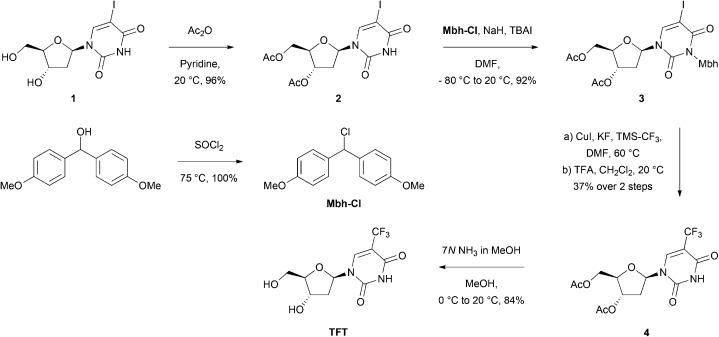
Synthesis of TFT *via* the iodinated nucleoside analogue **3**.

Trifluoromethylation of an iodinated uridine analogue has been reported only once, using copper powder and bis(trifluoromethyl)mercury.^36^ In that example, the iodinated precursor featured acetate protecting groups at the 3′- and 5′-alcohols and a *para*-methoxybenzyl protecting group at the ureido NH position. Since the ^18^F-trifluoromethylation procedure we planned to use was often incompatible with unprotected heteroatoms,^22^ we also synthesised a globally protected precursor (iodonucleoside analogue **3**, Scheme 1). We maintained the previously reported strategy by acetylating the 3′- and 5′-alcohols of 5-iodo-2′-deoxyuridine, but used the acid-labile 4,4′-dimethoxybenzhydryl (Mbh) protecting group at the ureido NH position, for easier deprotection during radiosynthesis.

Next, we aimed to trifluoromethylate the iodinated nucleoside analogue **3**, to gauge its suitability for the radioactive procedure, and to provide the reference compounds for product identification during the radiosynthesis. Inspired by a procedure used by Huiban *et al.*,^22^ we used the Ruppert–Prakash reagent to facilitate the trifluoromethylation. When combined with copper iodide and potassium fluoride, this reagent promotes the *in situ* generation of a CuCF_3_ intermediate,^37^ which is also the key intermediate of the radiochemical method.^22^ The iodinated nucleoside analogue **3** was successfully trifluoromethylated; however, we were unable to separate the product from the starting material using standard chromatographic purification. Isolation of a trifluoromethylated species became possible only after the removal of the Mbh protecting group, permitting access to **4**. The deprotection of the 3′- and 5′-alcohols using methanolic ammonia enabled access to TFT in an overall yield of 27%, thereby providing a synthetic route from an iodinated precursor using mild and stable reagents.

### Radiochemistry

Firstly, we evaluated the ^18^F-trifluoromethylation of precursor **3** (Table 1), since this compound contained no free heteroatoms that would potentially inhibit radiosynthesis. Product identity and radiochemical (RC) purity were determined using radio-HPLC. Radiochemical yields (RCYs) were derived from the crude reaction mixture unless stated otherwise (after quenching the reaction), and accounted for radiochemical purity. Using the conditions reported in the literature,^22^**[^18^F]-5** was formed in a RCY of just 11%, owing to the low RC purity of 14% (Table 1, entry 1). An increased RC purity of 28% was achieved through a six-fold increase in the proportions of chlorodifluoromethylacetate, copper iodide, and tetramethylenediamine (TMEDA), relative to the precursor (Table 1, entry 3), which was accompanied by an increase in RCY.

**Table 1:** Optimisation of ^18^F-trifluoromethylation of precursor **3**

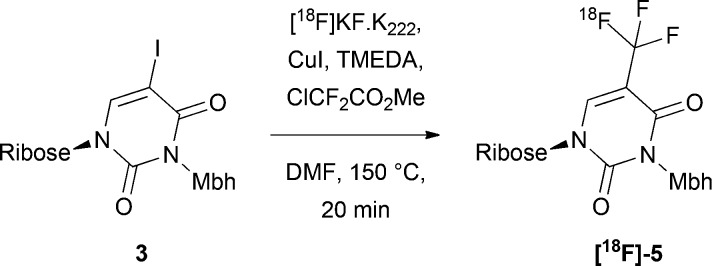			
Entry	Equivalents of ClCF_2_CO_2_Me, CuI and TMEDA^*a*^	RC purity (%)	RCY (%)
1	1.56	14	11
2	4	13	9
3	6	28	19

^*a*^Reagent equivalents are with respect to one equivalent of precursor.

Aiming to reduce the number of deprotection steps post-radiolabelling, we also studied the ^18^F-trifluoromethylation of the unprotected amide precursor **2** (Table 2). Using the reported procedure (involving manual addition of the liquid reagents to an azeotropically dried mixture of [^18^F]KF/K_222_ and CuI),^22^ no formation of **[^18^F]-4** was observed (Table 2, entry 1). The reaction was presumably disrupted by chelation of the copper at the free amide. Despite this, we were able to drive product formation by using six equivalents of chlorodifluoromethylacetate, copper iodide, and TMEDA, and obtained a RCY of 9% (Table 2, entry 3). Extending the reaction time beyond 20 minutes did not considerably improve the reaction outcome, but by generating [^18^F]KF/K_222_ from KHCO_3_ rather than K_2_CO_3_, the RCY was increased to 27% (Table 2, entry 6). Reducing the quantities of KHCO_3_ and K_222_ did not substantially hinder the reaction progression. Due to the heterogeneity of the reaction mixture, mixing was a critical parameter for reaction progress.

**Table 2:** Optimisation of ^18^F-trifluoromethylation of precursor **2**

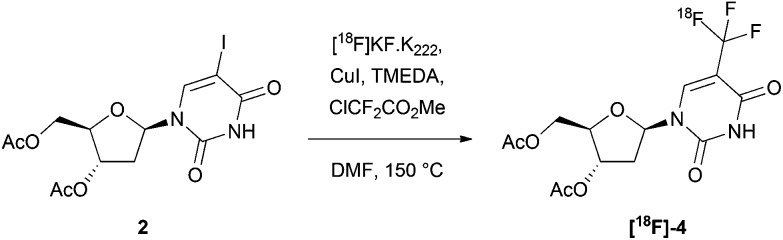							
Entry	Equivalents of ClCF_2_CO_2_Me and TMEDA^*a*^	Equivalents of CuI^*a*^ ^,^^*b*^	Equivalents of base and K_222_, respectively	Reaction time (minutes)	Base	RC purity (%)	RCY (%)
1	1.56	1.56	0.4, 0.5	20	K_2_CO_3_	0	0
2	4	4	0.4, 0.5	20	K_2_CO_3_	8	7
3	6	6	0.4, 0.5	20	K_2_CO_3_	14	9
4	6	6	0.4, 0.5	10	K_2_CO_3_	6	6
5	6	6	0.4, 0.5	30	K_2_CO_3_	15	11
6	6	6	0.4, 0.5	20	KHCO_3_	36	27
7	6	6	0.4, 0.5	30	KHCO_3_	24	16
8	6	6	0.24, 0.32	20	KHCO_3_	34	24
9	6	9	0.24, 0.32	20	KHCO_3_	46	27
10	4	6	0.24, 0.32	20	KHCO_3_	33	24

^*a*^Reagent equivalents are with respect to one equivalent of precursor.

^*b*^Copper iodide was added as a solid from the beginning of azeotropic fluoride drying.

While the inclusion of greater amounts of chlorodifluoromethylacetate, copper iodide, and TMEDA improved the RC purity, the discrepancy between the RCY and the RC purity also increased. During these reactions, we observed that a large proportion of activity was lost upon quenching, which could be partially trapped onto a solid-phase extraction (SPE) cartridge. We hypothesised that the volatile side product [^18^F]HCF_3_ may have formed, resulting from insufficient trapping of the reactive intermediate difluorocarbene with copper. To test this theory, we increased the proportion of copper iodide relative to chlorodifluoromethylacetate (Table 2, entries 9 and 10). Using 9 equivalents of copper iodide, we obtained our highest RC purity of 46% (Table 2, entry 9), however the RCY did not significantly improve. While the nature of the observed volatile side product remains unknown, the RCY yield and RC purity achieved was sufficiently high to commence adaption of the procedure for automation.

A fully automated radiosynthesis is desirable for clinical production in accordance with GMP guidelines, and because the entire process can be performed in an enclosed hot cell, far greater amounts of activity can be used. For these reasons, we automated the ^18^F-trifluoromethylation procedure using the Trasis AllInOne™ (AIO) PET tracer synthesiser (Fig. S1†). This is a cassette-based system, whereby the synthesiser manipulates a plastic manifold fitted with a sealed reactor, replaceable syringes, and solid phase extraction cartridges. Since the radiolabelling of precursor **2** would only require one deprotection procedure, the automated radiosynthesis was pursued only for precursor **2** (and not the protected precursor **3**).

We identified a number of features of the manual procedure that would not be compatible with automation. Firstly, the addition of solid copper iodide from the beginning of synthesis would not be possible, as the reaction vessel included with the AIO is irreversibly sealed. Secondly, to enable accurate transfer and to circumvent the need for stirring, all of the reagents used in the automated procedure would need to be prepared as homogenous solutions. Based on the optimum conditions for the ^18^F-trifluoromethylation of **2** established through manual radiosynthesis (Table 2, entry 9), the reaction components could not be sufficiently dissolved for the development of an efficient automated procedure. Three sets of conditions were tested, in which all reagents were soluble as a combined solution in DMF (Table 3). The best reaction outcome, in terms of both RCY and RC purity, was achieved using 4 equivalents each of copper iodide, TMEDA and ClCF_2_CO_2_Me, with a reaction concentration of 0.05 M with respect to precursor **2**.

**Table 3:** Optimisation of the reaction mixture composition for an efficient automated ^18^F-trifluoromethylation of precursor **2**

Entry	Equivalants of ClCF_2_CO_2_Me and TMEDA^*a*^	Equivalents of CuI^*a*^ ^,^^*b*^	Molarity^*c*^ (M)	RC purity^*d*^ (%)	RCY^*d*^ (%)
1	6	6	0.025	11	1
2	4	6	0.025	48	5
3	4	4	0.05	51	7

^*a*^Reagent equivalents are with respect to one equivalent of precursor.

^*b*^Copper iodide was added as a solution in DMF, also containing precursor **2**, ClCF_2_CO_2_Me and TMEDA.

^*c*^Molarity was based on precursor **2**.

^*d*^In this case, RC purity and RCY were calculated after partial purification using an HLB SPE cartridge.

The deprotection of intermediate **[^18^F]-4** was subsequently investigated (Table 4). Initially, methanolic ammonia was used to reflect the results achieved during non-radioactive synthesis (Scheme 1). Full conversion of **[^18^F]-4** was achieved after 15 minutes, however the RC purity was low due to the formation of an unknown polar side product. The reaction outcome improved considerably when a dilute, aqueous solution of sodium hydroxide was used instead. After 5 minutes of reaction, full conversion of **[^18^F]-4** to [^18^F]TFT was observed, with a RC purity of 56% even at room temperature.

**Table 4:** Relative RC purities of [^18^F]TFT achieved through deprotection of **[^18^F]-4**

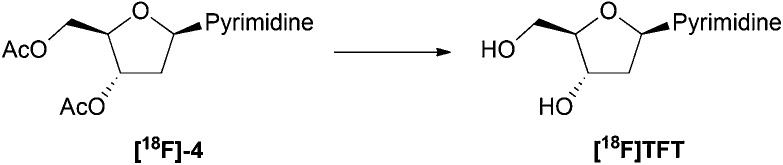			
Reagents	Time (min)	Temp (°C)	RC purity (%)
7.0 *N* NH_3_ in MeOH	15	40	15
0.2 M NaOH (aq.)	5	40	58
0.1 M NaOH (aq.)	5	20	56

**Scheme 2:**
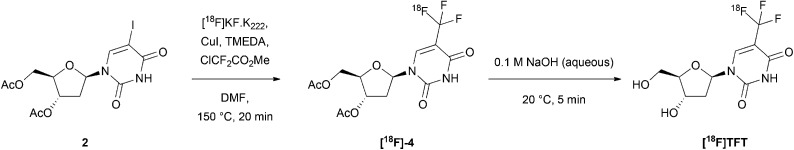
Automated radiosynthesis of [^18^F]TFT.

After semi-preparative RP-HPLC purification, [^18^F]TFT was isolated in an overall RCY of 3% ± 0.44 (*n* = 5), with >99% RC purity and a molar activity of 0.4 GBq μmol^–1^ ± 0.05 (*n* = 5) (Scheme 2). The total reaction time, from the delivery of aqueous [^18^F]fluoride to the hot cell, to the reformulation of [^18^F]TFT in H_2_O after purification, was approximately 90 minutes. While the molar activity was low, it was consistent with those reported by Huiban *et al.*^22^ [^18^F]TFT had a Log *D*_7.4_ value of –0.57 ± 0.01 (*n* = 3) and was >99% stable in water at ambient temperature for up to 5 hours (Table S1†).

### Biological evaluation

With access to an automated and reproducible method for [^18^F]TFT synthesis, we investigated the biological behaviour of [^18^F]TFT as a PET probe. Prior to *in vivo* experiments, we performed two separate *in vitro* tests that would generate some of the [^18^F]TFT metabolites expected to be observed *in vivo*, to aid us in RP-HPLC method development and product identification. It is well known that thymidine analogues (with the exception of FLT) are susceptible to enzymatic glycosidic bond cleavage,^38^ therefore [^18^F]TFT was incubated with human recombinant thymidine phosphorylase to observe the formation of the deglycosylated metabolite [^18^F]trifluorothymine by RP-HPLC (Table S2†). [^18^F]TFT was also incubated with HCT116 cell homogenates, in order to achieve the phosphorylation of the nucleoside analogue at the 5′-alcohol. Two radiolabelled [^18^F]TFT metabolites were observed on the HPLC chromatograms: [^18^F]trifluorothymine and a polar metabolite (eluting at the solvent front). Confirmation that the polar metabolite was a phosphorylated adduct was achieved by adding a phosphatase enzyme to the reaction components; reduction of the peak at the solvent front was observed, with rescue of the parent compound (Table S3†).

**Fig. 1:**
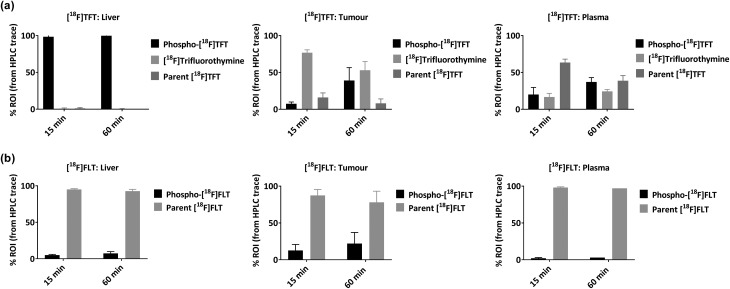
The radioactive metabolites of (a) [^18^F]TFT and (b) [^18^F]FLT observed *in vivo* using HCT116 tumour-bearing mice. The data were quantified using RP-HPLC, based on the area under the curve (region of interest) for (a) [^18^F]TFT (retention time (RT): 06:44 minutes:seconds (mm:ss)), [^18^F]trifluorothymine (RT: 05:27 mm:ss), and the phosphorylated adduct of [^18^F]TFT (RT: 03:50 mm:ss); and (b) [^18^F]FLT (RT: 07:59 mm:ss) and the phosphorylated adduct of [^18^F]FLT (RT: 03:42 mm:ss). Each experiment was performed in triplicate and the data were expressed as a percentage (mean ± SD).

We then investigated the radioactive metabolites of [^18^F]TFT produced *in vivo* using HCT116 tumour-bearing mice. We hypothesised that a large degree of [^18^F]TFT phosphorylation (leading to DNA incorporation) would improve PET signal retention in the tumour, whereas the extent of glycosidic bond cleavage would correlate with poor PET signal retention. For comparison, we also examined [^18^F]FLT. TFT and [^18^F]FLT are both mono-phosphorylated by TK1. However, their metabolic pathways differ thereafter, since [^18^F]FLT is not incorporated into DNA and is not susceptible to glycosidic bond cleavage. Liver, tumour and plasma samples were collected at 15 and 60 minutes post-injection of [^18^F]TFT or [^18^F]FLT, and the metabolites were analysed by RP-HPLC. The *in vivo* metabolic profile of [^18^F]TFT was consistent with the results from the *in vitro* assays, with the parent compound, the deglycosylated metabolite, and the phosphorylated adduct observed across all samples (Fig. 1). When the plasma was examined, we found that by 15 minutes the main circulating species was [^18^F]TFT. By 60 minutes, [^18^F]TFT, [^18^F]trifluorothymine and phospho-[^18^F]TFT were present in the plasma in comparable quantities. In the liver, the main radioactive compound detected was the phosphorylated product, as early as 15 minutes post-injection. This could indicate that the phosphorylation of [^18^F]TFT occurs in the liver, or that the liver facilitates clearance of [^18^F]TFT and the metabolites. In the tumour, small proportions of [^18^F]TFT and phospho-[^18^F]TFT were present after 15 minutes, with [^18^F]trifluorothymine as the major metabolite. By 60 minutes, an increased proportion of phospho-[^18^F]TFT was observed. When the same experiment was performed with [^18^F]FLT, the radiotracer was over 75% intact in all tissue and blood specimens, at both time points, indicating less of the desirable [^18^F]FLT phosphorylation, but superior metabolic stability (Fig. 1).

**Fig. 2:**
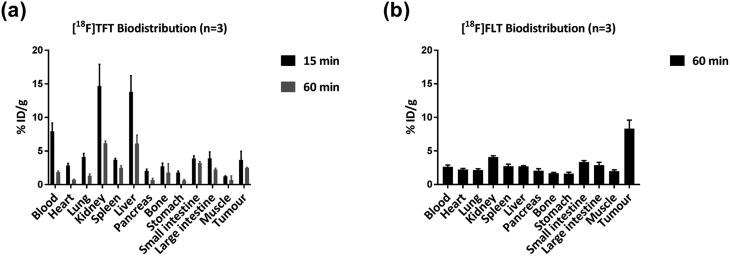
Biodistribution results gathered using HCT116 tumour-bearing mice, following administration of (a) [^18^F]TFT, recorded at 15 and 60 minutes post-injection, and (b) the [^18^F]FLT control (recorded at 60 minutes post-injection). Each experiment was performed in triplicate, and the data are reported as the mean %ID g^–1^ ± SD.

We also performed biodistribution and PET imaging following administration of [^18^F]TFT (∼10 MBq, 0.4 GBq μmol^–1^), using the same mouse model and time points (Fig. 2). At 15 minutes post-injection, the tumour uptake of [^18^F]TFT was 3.67 ± 1.28% injected dose per gram (%ID g^–1^) (Table S7†). An activity value of 7.94 ± 1.21 %ID g^–1^ was found in the blood at this time point, indicating that the tracer (and its metabolites) were still circulating and not completely cleared from the body. The high activity registered in the liver and kidneys suggests that the radiotracer is excreted *via* both the renal and the hepatobiliary systems. By 60 minutes post-injection, the tumour uptake of [^18^F]TFT was 2.49 ± 0.07 %ID g^–1^, with general improvement of the tumour : organ ratios (Table S8†).

Biodistribution data were also collected at 60 minutes post-injection of [^18^F]FLT (∼10 MBq, 0.1 GBq μmol^–1^) (Fig. 2). [^18^F]FLT was produced with a similar molar activity to [^18^F]TFT, for a more reliable comparison of biodistribution results. At this time point, the tumour uptake of [^18^F]FLT was 8.32 ± 1.28 %ID g^–1^ (Table S7†), and no notable radioactivity was observed in any other tissue, hinting at rapid excretion of the tracer from the body. The markedly higher uptake of [^18^F]FLT in the tumour compared with [^18^F]TFT is most likely due to the improved metabolic stability of [^18^F]FLT, regarding glycosidic bond cleavage for example. Notably, the production of [^18^F]FLT with low molar activity did not prevent tumour uptake, suggesting that the low molar activity associated with the ^18^F-trifluoromethylation procedure would not limit the potential of [^18^F]TFT as a PET probe.

**Fig. 3:**
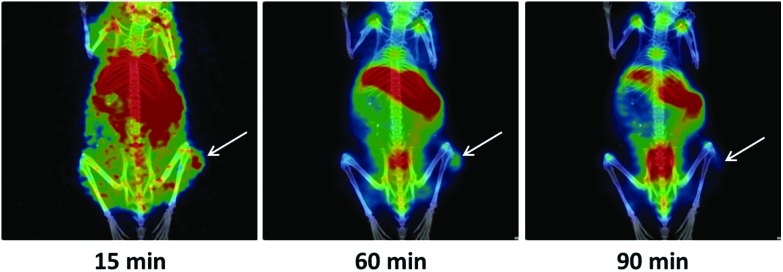
Representative PET/CT images selected from a 90-minute dynamic PET scan of a mouse bearing a HCT116-derived tumour, following the administration of [^18^F]TFT. The white arrows indicate the tumour.

To visualise and quantify the tumour uptake of [^18^F]TFT over time, 90-minute dynamic PET scans were acquired. The tumour was detected as early as 15 minutes post-injection (Fig. 3). By 60 minutes post-injection, the tumour could be more clearly identified, with clearance of much of the background activity that was observed at 15 minutes. On the other hand, by 90 minutes post-injection, little activity remained in the tumour (Fig. 3, Fig. S10†), and the bone uptake was increasingly prominent. It is known that proliferation imaging agents, including [^18^F]FLT, can localise in bone marrow.^32^ From our biodistribution data, however, the tumour : bone %ID g^–1^ ratio for [^18^F]FLT was considerably improved with respect to [^18^F]TFT (Table S8†). These data, coupled with the loss of [^18^F]TFT from the tumour over time, suggest that fluorine-18 release from [^18^F]TFT is a more likely explanation for the bone uptake, rather than localisation of [^18^F]TFT in the bone marrow. Furthermore, defluorination can be directly connected to the mode of action of TFT. Although DNA incorporation of tri-phosphorylated TFT is considered the main mechanism by which it exerts anticancer properties,^39^,^40^ mono-phosphorylated TFT also covalently binds to and inhibits thymidylate synthase (TS).^41^ Several reports have determined that this covalent linkage results in a stepwise loss of the three fluorine substituents of TFT,^42^–^44^ which in the case of [^18^F]TFT, would result in bone uptake of free [^18^F]fluoride in the PET scans.

It is now clear that while tumour uptake of [^18^F]TFT can be observed in a PET image up to 60 minutes post-injection, [^18^F]TFT defluorination becomes a prominent feature of the imaging data at later time points, which may limit its use as a proliferation imaging agent. Nonetheless, the [^18^F]TFT PET probe may provide a new method of elucidating drug mechanism of action. Using biodistribution studies, metabolite analysis and PET imaging, we observed thymidine phosphorylase-mediated glycosidic bond cleavage, kinase-mediated phosphorylation, and thymidylate synthase-mediated defluorination. These experiments could be used as a platform to enable further quantitative analyses of the biological processes associated with TFT administration.

## Conclusions

In this work, we have demonstrated the effectiveness of an optimised ^18^F-trifluoromethylation procedure to radiolabel a nucleoside substrate. Furthermore, we successfully adapted the procedure for automation, to provide a clinically-relevant method for the large-scale production of ^18^F-trifluoromethylated radiotracers. This method was tested for the synthesis of [^18^F]trifluridine, which is the radiolabelled derivative of the anticancer agent 5-trifluoromethyl-2′-deoxyuridine, and we performed preliminary investigations into its biological behaviour. Our data suggest that the low molar activity associated with the radiochemistry is not a barrier to the performance of [^18^F]TFT as a PET probe. However, we observed that in mice the retention of [^18^F]TFT in the tumour decreased over time, probably as a consequence of glycosidic bond cleavage and [^18^F]fluoride elimination. To serve as a proliferation imaging agent, further investigation into the administration of [^18^F]TFT (through co-administration with a thymidine phosphorylase inhibitor, for example) is required to improve its pharmacokinetics, metabolism, and the resulting image quality. Because of the ability to gain pharmacokinetic data in real-time, the development of [^18^F]TFT can provide a useful tool for the elucidation of drug mechanism of action *in vivo*.

## Experimental

### Abbreviations

Ac: acetyl; Bq: Becquerel; cc: centimetres cubed; DMF: *N*,*N*-dimethylformamide; Et: ethyl; K_222_: Kryptofix®222; Mbh: 4,4′-(methylene)bis(methoxybenzene); Me: methyl; RP-HPLC: reverse-phase high performance liquid chromatography; TBAI: tetrabutylammonium iodide; TFA: trifluoroacetic acid; TMEDA: tetramethylenediamine; TMS: trimethylsilyl.

### Chemistry

#### General methods

All reagents and anhydrous solvents were obtained from commercial suppliers (Carbosynth UK, Fisher Scientific UK, Sigma Aldrich UK) and used without further purification. Reactions were performed in oven-dried glassware under an atmosphere of nitrogen unless otherwise stated. Thin layer chromatography (TLC) was performed on pre-coated aluminium sheets of silica (60 F_254_, Merck, UK) and visualised by short-wave UV light and treatment with KMnO_4_ staining. Semi-automated flash column chromatography was carried out on a Biotage Isolera purification system, using Biotage SNAP cartridges (10 g, 25 g, 50 g, 100 g, 340 g). ^1^H NMR spectra were recorded at 500 MHz on a Bruker Avance-AMX500 using an internal deuterium lock. ^13^C NMR spectra were recorded at 126 MHz on a Bruker Avance-AMX500 using an internal deuterium lock. All chemical shifts were measured in parts per million (ppm) relative to tetramethylsilane (*δ* = 0) and are referenced to a residual solvent peak. LCMS and HRMS analysis was performed on an Agilent 1200 series HPLC and diode array detector coupled to a 6520 Quadrupole-Time of flight mass spectrometer with dual multimode APCI/ESI source. Analytical separation was carried out at 30 °C in a Merck Purospher STAR column (RP-18e, 30 × 4 mm) using a flow rate of 1.5 mL min^–1^ in a 4 min gradient elution with detection at 254 nm. The mobile phase was a mixture of methanol and water, both containing formic acid at 0.1% (v/v). The following reference masses were used for HRMS analysis: caffeine [M + H]^+^ 195.087652; (hexakis(1*H*,1*H*,3*H*-tetrafluoropentoxy)phosphazene [M + H]^+^ 922.009798 and hexakis(2,2-difluoroethoxy)phosphazene [M + H]^+^ 622.02896 or reserpine [M + H]^+^ 609.280657).

### Synthesis of TFT, Mbh-Cl, and compounds **2–4**

#### 5-Iodo-2′-deoxyuridine-3′,5′-diacetate (**2**)

To a solution of 2′-deoxy-5-iodouridine (1.0 g, 2.8 mmol) in pyridine (7 mL) was added Ac_2_O (1.1 mL, 11.3 mmol) and the reaction mixture was stirred at room temperature for 16 hours. The reaction mixture was concentrated *in vacuo*, and the residue was dissolved in toluene (30 mL) and co-evaporated *in vacuo* to yield the product as a white solid (1.2 g, 96%). The product was used in the next reaction step with no further purification. The data were in agreement with the published values for this structure.^45^^1^H NMR (500 MHz, CDCl_3_): *δ*_H_ = 8.75 (s, 1H), 7.97 (s, 1H), 6.28 (dd, *J* = 8.2 Hz, *J* = 5.7 Hz, 1H), 5.23 (dt, *J* = 6.5 Hz, *J* = 2.3 Hz, 1H), 4.41 (dd, *J* = 12.3 Hz, *J* = 3.2 Hz, 1H), 4.33 (dd, *J* = 12.3 Hz, *J* = 2.9 Hz, 1H), 4.30 (dd, *J* = 5.5 Hz, *J* = 2.9 Hz, 1H), 2.54 (ddd, *J* = 14.2 Hz, *J* = 5.7 Hz, *J* = 2.1 Hz, 1H), 2.21 (s, 3H), 2.20–2.16 (m, 1H), 2.12 (s, 3H); ^13^C NMR (126 MHz, CDCl_3_): *δ*_C_ = 170.3, 170.0, 159.3, 149.5, 143.6, 85.4, 82.6, 74.0, 68.7, 63.8, 38.2, 21.0, 20.8; HRMS (ES+) calculated 460.9816 for C_13_H_15_IN_2_O_7_Na, observed 460.9824 [M + Na]^+^.

#### 4,4′-(Chloromethylene)bis(methoxybenzene) (**MBh-Cl**)

SOCl_2_ (3.5 mL, 45.1 mmol) was added slowly to 4,4′-dimethoxybenzhydrol (1.0 g, 4.1 mmol) and the mixture heated to reflux and stirred for 2.5 hours. The reaction mixture was then cooled to room temperature and concentrated *in vacuo* to yield the crude product as a pink solid. This was used directly in the next step with no further purification (1.1 g, 100%). The data were in agreement with the published values for this structure.^46^^1^H NMR (500 MHz, CDCl_3_): *δ*_H_ = 7.34–7.33 (m, 2H), 7.32–7.31 (m, 2H), 6.89–6.88 (m, 2H), 6.87–6.86 (m, 2H), 6.12 (s, 1H), 3.80 (s, 6H); ^13^C NMR (126 MHz, CDCl_3_): *δ*_C_ = 159.3, 133.5, 129.0, 113.8, 64.2, 55.3; HRMS (ES+) no ionisation.

#### (N^3^-*para*-(Methylene)bis(methoxybenzene))-5-iodo-2′-deoxyuridine-3′,5′-diacetate (**3**)

To a solution of **2** (1.2 g, 2.7 mmol) in DMF (20 mL) at –60 °C was added NaH (60%) (159 mg, 4.0 mmol) and the resulting solution stirred for 30 minutes at this temperature. TBAI (100 mg, 0.3 mmol) was added followed by **Mbh-Cl** (1.1 g, 4.2 mmol) in DMF (6.5 mL) and the solution was warmed to room temperature and stirred for 16 hours. The reaction mixture was quenched with H_2_O (200 mL) and extracted with EtOAc (200 mL). The organic layer was washed with H_2_O : brine (1 : 1, 2 × 200 mL), dried over MgSO_4_ and concentrated *in vacuo*. The crude material was purified using flash silica column chromatography (20–50% EtOAc in cyclohexane) to yield the product as a white foam (1.6 g, 92%). ^1^H NMR (500 MHz, CDCl_3_): *δ*_H_ = 7.98 (s, 1H), 7.31–7.29 (m, 4H), 7.26 (s, 1H), 6.86–6.83 (m, 4H), 6.24 (dd, *J* = 8.2 Hz, *J* = 5.6 Hz, 1H), 5.19 (dt, *J* = 6.5 Hz, *J* = 2.3 Hz, 1H), 4.38 (dd, *J* = 12.3 Hz, *J* = 3.3 Hz, 1H), 4.32 (dd, *J* = 12.3 Hz, *J* = 3.0 Hz, 1H), 4.26 (dd, *J* = 6.5 Hz, *J* = 3.0 Hz, 1H), 3.80 (s, 3H), 3.79 (s, 3H), 2.50 (ddd, *J* = 14.2 Hz, *J* = 5.6 Hz, *J* = 2.1 Hz, 1H), 2.20 (s, 3H), 2.12–2.07 (m, 1H), 2.07 (s, 3H); ^13^C NMR (126 MHz, CDCl_3_): *δ*_C_ = 170.5, 170.4, 159.8, 159.1, 150.2, 142.4, 130.2, 113.8, 86.3, 82.7, 74.3, 69.2, 64.0, 60.6, 55.5, 38.6, 21.4, 21.0. Note: Two carbon signals are overlapping; HRMS (ES+) calculated 687.0810 for C_28_H_29_IN_2_O_9_Na, observed 687.0803 [M + Na]^+^.

#### 5-Trifluoromethyl-2′-deoxyuridine-3′,5′-diacetate (**4**)

To a solution of **3** (300 mg, 0.45 mmol), CuI (103 mg, 0.54 mmol) and KF (39 mg, 0.68 mmol) in DMF (0.9 mL) at room temperature was added TMS-CF_3_ (80 μL, 0.54 mmol). After stirring for 16 hours at 60 °C, the reaction mixture was poured into H_2_O (20 mL) and extracted into EtOAc (2 × 20 mL). The combined organic layers were dried over MgSO_4_ and concentrated *in vacuo*. The crude residue was purified by flash silica column chromatography (20–40% EtOAc in cyclohexane). To the residue (a mixture of trifluoromethyl-**5** and iodo-**3**) was added CH_2_Cl_2_ (5.4 mL) and TFA (0.6 mL), and the reaction mixture was stirred at room temperature for 16 hours. The reaction mixture was concentrated *in vacuo* and purified by flash silica column chromatography (30–40% EtOAc in cyclohexane) to yield the product as a brown foam (63 mg, 37%). The deprotected iodonucleoside analogue **2** was also recovered (40 mg, 20%). The data were in agreement with the published values for structure **4**.^47^^1^H NMR (500 MHz, CDCl_3_): *δ*_H_ = 8.76 (br. s, 1H), 8.09 (s, 1H), 6.27 (dd, *J* = 8.1 Hz, *J* = 5.6 Hz, 1H), 5.23 (dt, *J* = 6.5 Hz, *J* = 2.1 Hz, 1H), 4.43 (dd, *J* = 11.8 Hz, *J* = 2.6 Hz, 1H), 4.35–4.30 (m, 2H), 2.63 (ddd, *J* = 14.3 Hz, *J* = 5.6 Hz, *J* = 2.1 Hz, 1H), 2.17 (ddd, *J* = 14.4 Hz, *J* = 8.1 Hz, *J* = 6.5 Hz, 1H), 2.12 (s, 3H), 2.10 (s, 3H); ^13^C NMR (126 MHz, CDCl_3_): *δ*_C_ = 170.2, 170.0, 157.9, 149.0, 139.9 (q, ^*3*^*J*_C–F_ = 5.9 Hz), 121.6 (q, *J*_C–F_ = 270.2 Hz), 105.7 (q, ^*2*^*J*_C–F_ = 33.4 Hz), 85.9, 83.0, 73.9, 63.6, 38.5, 20.7, 20.4; ^19^F NMR (500 MHz, CDCl_3_): *δ*_F_ = –63.5; HRMS (ES+) calculated 403.0831 for C_14_H_15_F_3_N_2_O_7_Na, observed 403.0815 [M + Na]^+^.

#### 5-Trifluoromethyl-2′-deoxyuridine (**TFT**)

To a solution of **4** (60 mg, 0.16 mmol) in MeOH (3.2 mL) at 0 °C was added 7N NH_3_ in MeOH (6.2 mL, 43.6 mmol) and the reaction mixture was stirred at room temperature for 16 hours. The reaction mixture was concentrated *in vacuo* and the crude material purified using flash silica column chromatography (0–10% MeOH in CH_2_Cl_2_) to yield the product as a white solid (40 mg, 84%). The data were in agreement with the published values for this structure.^34^^1^H NMR (500 MHz, MeOD): *δ*_H_ = 8.80 (br. s, 1H), 6.25 (t, *J* = 6.2 Hz, 1H), 4.42 (dt, *J* = 6.2 Hz, *J* = 4.0 Hz, 1H), 3.97 (dd, *J* = 6.2 Hz, *J* = 2.9 Hz, 1H), 3.84 (dd, *J* = 11.9 Hz, *J* = 2.9 Hz, 1H), 3.75 (dd, *J* = 11.9 Hz, *J* = 2.9 Hz, 1H), 2.37 (ddd, *J* = 13.7, *J* = 6.2 Hz, *J* = 4.3 Hz, 1H), 2.27 (dt, *J* = 13.7 Hz, *J* = 6.2 Hz, 1H); ^13^C NMR (126 MHz, MeOD): *δ*_C_ = 161.4, 151.5, 144.0 (q, ^3^*J*_C–F_ = 6.0 Hz), 124.1 (q, *J*_C–F_ = 268.8 Hz), 105.5 (q, ^2^*J*_C–F_ = 32.9 Hz), 89.5, 87.7, 71.9, 62.3, 42.3; HRMS (ES+) calculated 319.0509 for C_10_H_11_F_3_N_2_O_5_Na, observed 319.0492 [M + Na]^+^.

### Radiochemistry

#### General methods

[^18^F]Fluoride was produced by a GE PETrace cyclotron by 16 MeV irradiation of enriched [^18^O]H_2_O target, supplied by Alliance Medical Radiopharmacy Ltd (Warwick, UK) and delivered to a dispensing hot cell in approximately 2 mL of water. [^18^F]Fluoride was used without further purification. The automated radiosynthesis platform used in the study was the Trasis AllInOne (Trasis, Belgium). Analytical and semi-preparative RP-HPLC were carried out on an Agilent 1260 quaternary pump system (Agilent Technologies) equipped with a Lablogic radioTLC/HPLC detector and a Lablogic NaI/PMT radiodetector. Oasis HLB SPE cartridges were purchased from Waters (Elstree, UK) and were conditioned using CH_3_CN (6 mL) and water (10 mL). Radioactivity was measured in a CRC -55tR dose calibrator (Capintec, Inc). Product identity and radiochemical (RC) purity were determined using radio-HPLC. RCY was derived from the crude reaction mixture unless stated otherwise (after quenching the reaction), and accounted for radiochemical purity. All RCYs quoted are decay-corrected to the activity of the aqueous [^18^F]fluoride used at the start of the reaction.
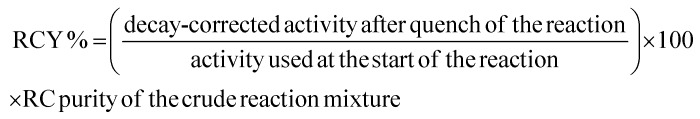


#### HPLC conditions

Intermediate **[^18^F]-4** was analysed on a Luna C18 column, 4.6 × 150 mm, 5 μM (Phenomenex) using *isocratic method 1*: eluant A H_2_O, 65%; eluant B CH_3_CN, 35%; flow rate 1 mL per minute. **[^18^F]TFT** was analysed using the same column type and *isocratic method 2*: eluant A H_2_O, 90%; eluant B EtOH, 10%; flow rate 1 mL per minute. **[^18^F]TFT** was purified by semi-preparative RP-HPLC using an Ultracarb™ ODS(30) C18 column, 10 × 250 mm, 7 μM (Phenomenex) and *isocratic method 3*: eluant A H_2_O, 88%; eluant B EtOH, 12%; flow rate 1 mL per minute. For *in vitro* experiments and *in vivo* metabolite analysis, **[^18^F]TFT** and the resultant metabolites were monitored using a μBondapak C18 column, 7.8 × 300 mm, 10 μm, 125 Å (Waters, UK) and *isocratic method 4*: eluant A H_2_O, 90%; eluant B EtOH, 10%; flow rate 3 mL min^–1^.

#### Optimised procedures for the manual radiosyntheses of intermediates **[^18^F]-4** and **[^18^F]-5**

Aqueous [^18^F]fluoride (50–150 MBq) was added to a capped V-vial containing a magnetic stirrer bar, CuI (9 eq. for **[^18^F]-4**; 6 eq. for **[^18^F]-5**) Kryptofix-222 (4.5 mg, 0.012 mmol), KHCO_3_ (0.9 mg in 100 μL H_2_O, 0.009 mmol) and CH_3_CN (500 μL) and dried under nitrogen at 100 °C. The V-vial contents were redissolved in CH_3_CN (500 μL) and dried under nitrogen twice more until no water remained. A solution of precursor **2** or **3** (0.037 mmol), TMEDA (33 μL, 0.222 mmol) and ClCF_2_CO_2_Me (23 μL, 0.222 mmol) in DMF (300 μL) was added to the V-vial containing the [^18^F]KF/K_222_/CuI and the reaction mixture was heated to 150 °C with stirring for 20 minutes. After fitting the V-vial was fitted with an outlet (or an SPE cartridge for trapping of volatiles), the reaction was quenched with H_2_O (500 μL). Without purification, the RC purity of **[^18^F]-4** was 46%, resulting in a RCY of 27%; the RC purity of **[^18^F]-5** was 28%, resulting in a RCY of 19%. The total reaction time in both cases was approximately 40 minutes.

#### Optimised automated radiosynthesis of **[^18^F]TFT 5**

On a Trasis AllInOne system, aqueous [^18^F]fluoride (∼12 GBq) was trapped onto a QMA-SPE cartridge (Waters, UK) pre-conditioned with KHCO_3_ (4 mL, 1.5 mg mL^–1^) and H_2_O (4 mL). The cartridge was blown dry under nitrogen. The [^18^F]fluoride was eluted with a 9 : 1 mixture of CH_3_CN : H_2_O containing Kryptofix 222 (2.75 mg) and KHCO_3_ (0.55 mg) into the reactor and was evaporated to dryness. After cooling to room temperature, a mixture of CuI (17.5 mg, 0.092 mmol), TMEDA (14 μL, 0.092 mmol), ClCF_2_CO_2_Me (10 μL, 0.092 mmol) and precursor **2** (10 mg, 0.023 mmol) in DMF (500 μL) was added and heated to 150 °C for 15 minutes. The reactor was cooled to room temperature and CH_3_CN (1 mL) was added. The reaction mixture was withdrawn into a syringe containing H_2_O (10 mL) and the reactor was rinsed. The solution containing intermediate **[^18^F]-4** and the washings were loaded onto a HLB-SPE cartridge, and the cartridge was dried under nitrogen. The product was eluted from the cartridge in CH_3_CN and transferred back to the reactor. After evaporating CH_3_CN by heating at 85 °C under nitrogen flow/vacuum, the reactor was cooled to room temperature. 0.1 M NaOH (1 mL) was added to the reactor and the reaction was carried out at room temperature with nitrogen bubbling through for 5 minutes. NaH_2_PO_4_ buffer (1 mL) was added to neutralise the solution, then the mixture was diluted with H_2_O and loaded onto the semipreparative HPLC system *via* a 0.2 μm Millex filter (Millipore, Billerica, MA, USA). **[^18^F]TFT 5** was purified using *isocratic method 3*, collected in a reformulation vial then loaded onto two HLB cartridges connected in series to concentrate it. The product was manually eluted with ethanol (1 mL), which was evaporated. The final product was then redissolved in the required volume of H_2_O. The RCY of the isolated product was 3% ± 0.44 (*n* = 5), with a radiochemical purity >99%, and a molar activity of 0.4 GBq μmol^–1^. The total reaction time, from delivery of aqueous [^18^F]fluoride to the hot cell, to the final reformulated product, was approximately 90 minutes.

### *In vivo* experiments

#### Cells and cell culture

All media and reagents for cell culture were purchased from Life Technology, Paisley, UK, unless otherwise stated. HCT116 colorectal carcinoma cells (from American Type Culture Collection) were cultivated in Dulbecco's Modified Eagle Medium (DMEM with 3.97 mM glutamine, 25 mM d-glucose, without sodium pyruvate) supplemented with non-essential amino acids, 10% heat-inactivated foetal bovine serum, 100 U mL^–1^ penicillin and 100 μg mL^–1^ streptomycin. Cells were incubated in an incubator at 37 °C in a humidified atmosphere with 5% CO_2_. The cells were shown to be mycoplasma free using a PCR-based assay (Surrey Diagnostics Ltd) and were authenticated in our laboratory by short tandem repeat (STR) profiling.

#### HCT116 (colorectal carcinoma) tumour model

All experiments were performed in compliance with licenses issued under the UK Animals (Scientific Procedures) Act 1986 and following local ethical review. Studies were compliant with the United Kingdom National Cancer Research Institute Guidelines for Animal Welfare in Cancer Research.^48^

#### Metabolite analysis

HCT116 cells (5 × 10^6^) were subcutaneously injected into the flank of NCr female nude mice. When the tumours reached ∼100 mm^3^ (between 10–15 days post-inoculation), the mice were injected *via* the lateral tail vein with the appropriate radiotracer in sterile saline solution (∼10 MBq). At 15 or 60 minutes post-injection, the mice were sacrificed by exsanguination *via* cardiac puncture under general anaesthesia (isoflurane inhalation). Liver, tumour, and blood samples were collected and snap-frozen in dry ice into appropriate containers: metal bead kit reinforced homogenisation tube (2.8 mm 2 mL, Stretton Scientific, UK) for liver and tumour samples; Eppendorf LoBind microcentrifuge tube (Sigma Aldrich, UK) containing heparin for blood samples. All tissue samples were thawed on ice prior to processing. Blood samples: after centrifugation (2000*g* for 5 minutes, 4 °C), the plasma was collected and added to LoBind microcentrifuge tubes containing ice-cold CH_3_CN (1 mL). Liver and tumour samples: after adding CH_3_CN (600 μL), the samples were homogenised at 10 °C using a PRECELLYS® 24 tissue homogeniser fitted with a cryolys (liver: 3 × 20 s; tumour: 6 × 20 s). All processed samples (blood, liver and tumour) were then centrifuged (12 000*g* for 5 minutes, 4 °C), and the supernatant was collected and evaporated to dryness. Prior to RP-HPLC analysis, the residues were diluted with H_2_O/acetonitrile mixture (9 : 1, 350 μL) and passed through a Millex 0.2 μm filter (Millipore, Billerica, MA, USA). The data were quantified using RP-HPLC, based on the area under the curve (region of interest) for each peak observed in the radio-chromatogram. Each experiment was performed in triplicate and the data were expressed as a percentage (mean ± SD).

#### Biodistribution studies

HCT116 tumour-bearing NCr female nude mice (as prepared for the metabolite analysis procedure) were injected *via* the lateral tail vein with the appropriate radiotracer in sterile saline solution (∼10 MBq). At 15 or 60 minutes post-injection, the mice were sacrificed by exsanguination *via* cardiac puncture under general anaesthesia (isoflurane inhalation). The appropriate tissues were then excised, weighed and the radioactivity was measured using a 2480 WIZARD^2^ automatic gamma counter (PerkinElmer, UK). Each experiment was performed in triplicate and data were expressed as the mean percentage of the injected dose per gram of tissue (%ID g^–1^) ± SD.

#### Dynamic PET imaging

PET/CT images were acquired using an Albira PET/SPECT/CT imaging system (Bruker). One HCT116 tumour-bearing NCr mouse was administered [^18^F]TFT (∼5 MBq) by intravenous tail vein injection. Approximately 5 minutes prior to imaging, the mouse was anesthetised using an isoflurane/O_2_ mixture (1.5–2.0% v/v) and placed prone in the centre of the scanner. Whole body dynamic PET data were acquired for a total duration of 90 minutes with a total of 60 frames (30 × 10 seconds (s), 20 × 30 s, 5 × 5 min and 5 × 10 min), followed by CT acquisition. The PET images were reconstructed using an MLEM algorithm (12 iterations) with a voxel size of 0.5 × 0.5 × 0.5 mm^3^. Whole body standard high resolution CT scans were performed with the X-ray tube set at a voltage of 45 kV, a current of 400 μA, 250 projections (1 s per projection), and a voxel size of 0.5 × 0.5 × 0.5 mm^3^. The CT images were reconstructed using a FBP algorithm. Image analysis was performed using the PMOD software package (PMOD Technologies Ltd, CH) and quantification was achieved by drawing a volume of interest (VOI) over the tumour using a 50% threshold. The mean counts were recorded and subsequently converted into kBq cc^–1^.

## Conflicts of interest

The authors declare that they have no conflict of interest.

## Supplementary Material

Supplementary information
