# Supplementary material for: Radiosynthesis of the anticancer nucleoside analogue Trifluridine using an automated 18F-trifluoromethylation procedure
Source: Org Biomol Chem. 2018 Apr 9;16(16):2986–96. doi: 10.1039/c8ob00432c (PMC5944245; doi:10.1039/c8ob00432c)
Supplement: Supplementary file 1 [file OB-016-C8OB00432C-s001.pdf]

Electronic Supplementary Material (ESI) for Organic & Biomolecular Chemistry.
This journal is © The Royal Society of Chemistry 2018

Supplementary information

1. NMR spectra **2**

1.1. ^{1}H NMR: Compound **2** 2

1.2. ^{13}C NMR: Compound **2** 3

1.3. ^{1}H NMR: Compound **3** 4

1.4. ^{13}C NMR: Compound **3** 5

1.5. ^{1}H NMR: Compound **4** 6

1.6. ^{13}C NMR: Compound **4** 7

1.7. ^{1}H NMR: Compound **5** 8

1.8. ^{13}C NMR: Compound **5** 9

1.9. ^{1}H NMR: Compound **7** 10

1.10. ^{13}C NMR: Compound **7** 11

2. Radiochemistry **12**

2.1. [^{18}F]TFT automation 12

2.2. Characterisation of [^{18}F]-**4** and [^{18}F]TFT **5** by radio-HPLC 12

2.3. Determination of [^{18}F]TFT molar activity 13

2.4. [^{18}F]FLT radiosynthesis procedure 14

3. LogD$_{7.4}$ **14**

4. Radiotracer stability in H_2O at ambient temperature **14**

5. *In vitro* thymidine phosphorylase assay **16**

5.1. Procedure 16

5.2. HPLC data 16

5.3. Results 17

6. *In vitro* cell homogenates assay **18**

6.1. Procedure 18

6.2. HPLC data 19

6.3. Results 19

7. *In vivo* metabolite analysis **20**

7.1. HPLC data 20

7.2. Results 20

8. Biodistribution studies **21**

9. Dynamic PET scanning **22**

1. NMR spectra

1.1. ^{1}H NMR compound **2**

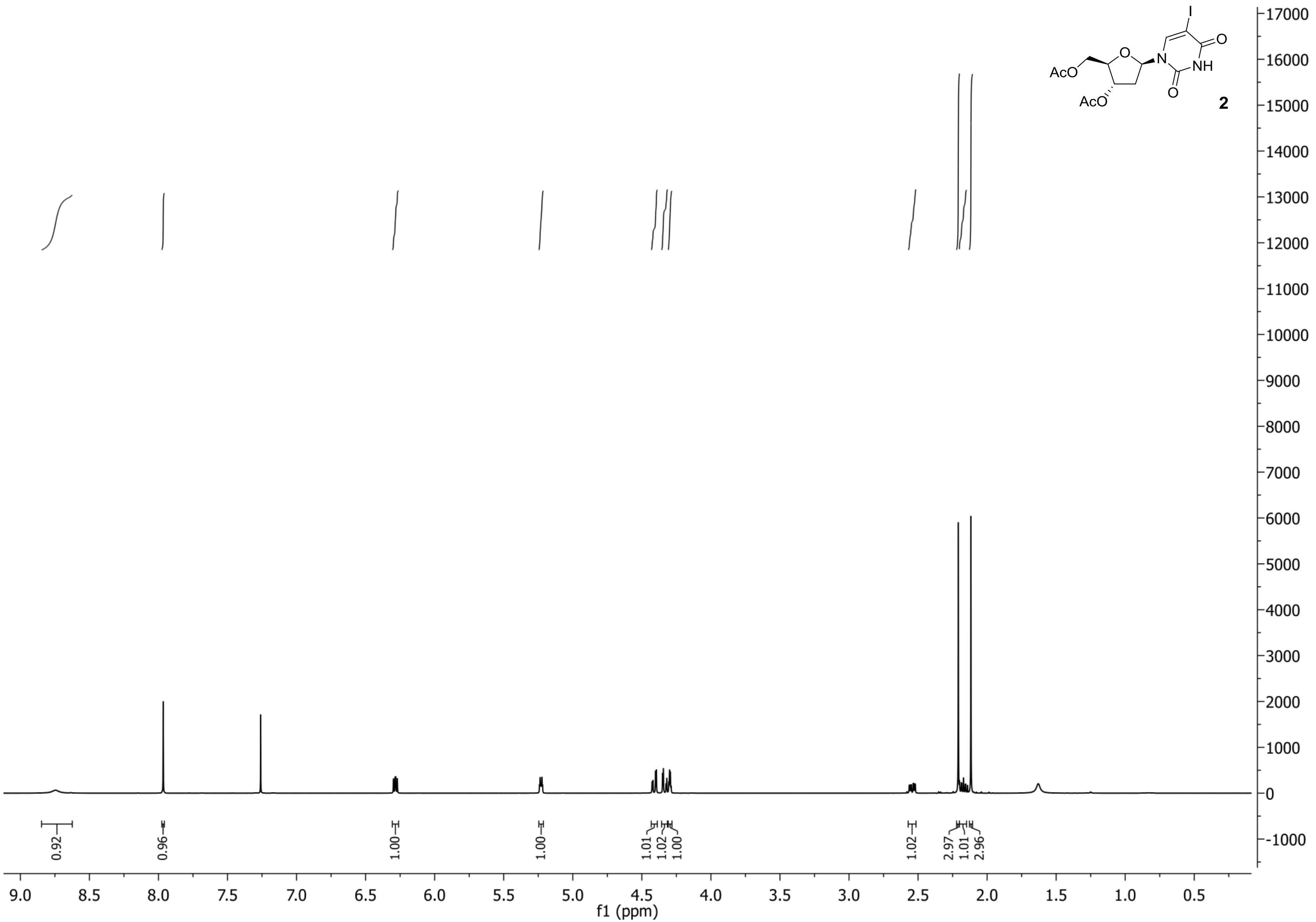

1.2. ^{13}C NMR compound **2**

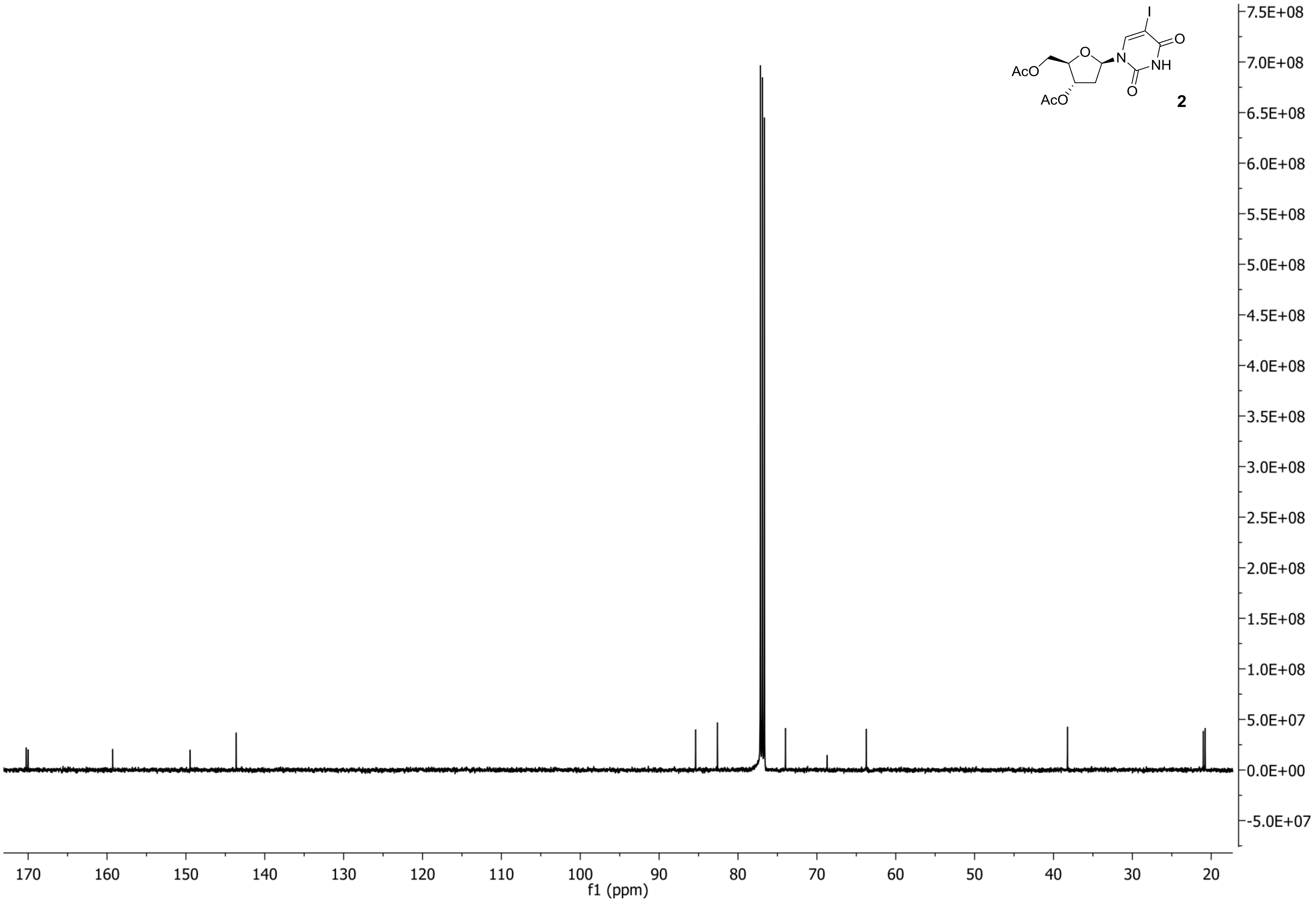

1.3. ^{1}H NMR compound **3**

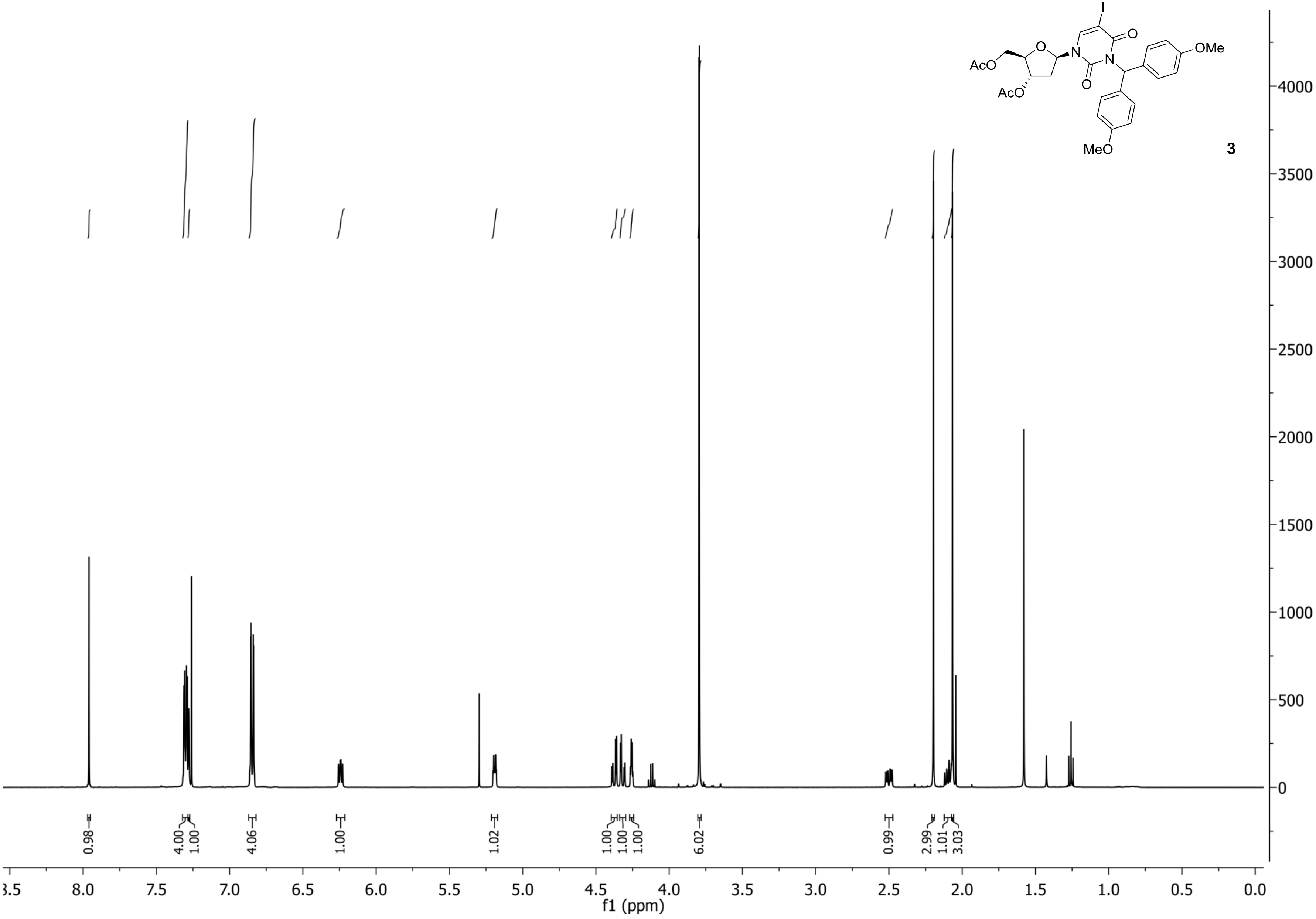

3

1.4. ^{13}C NMR compound **3**

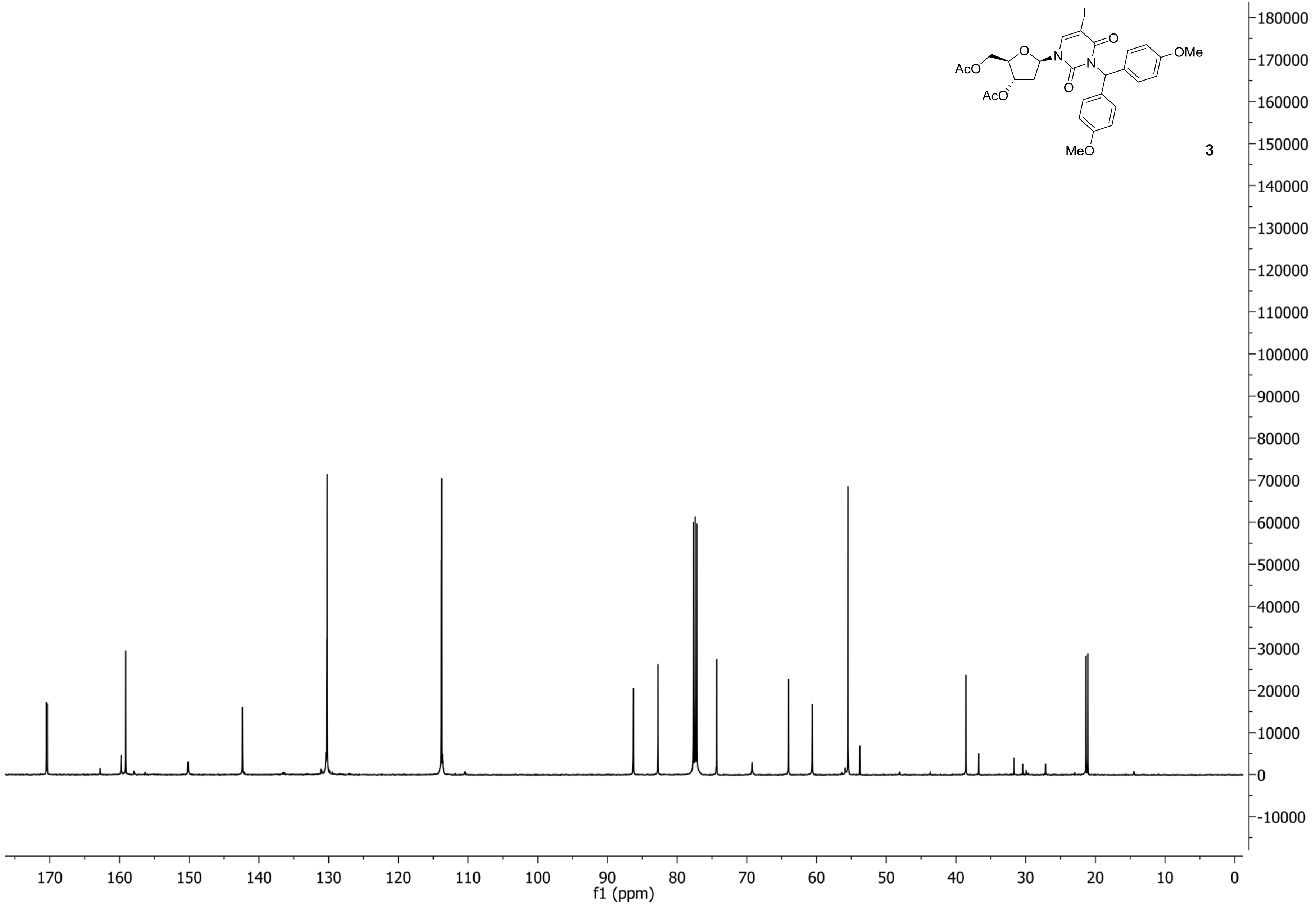

1.5. ^{1}H NMR compound **4**

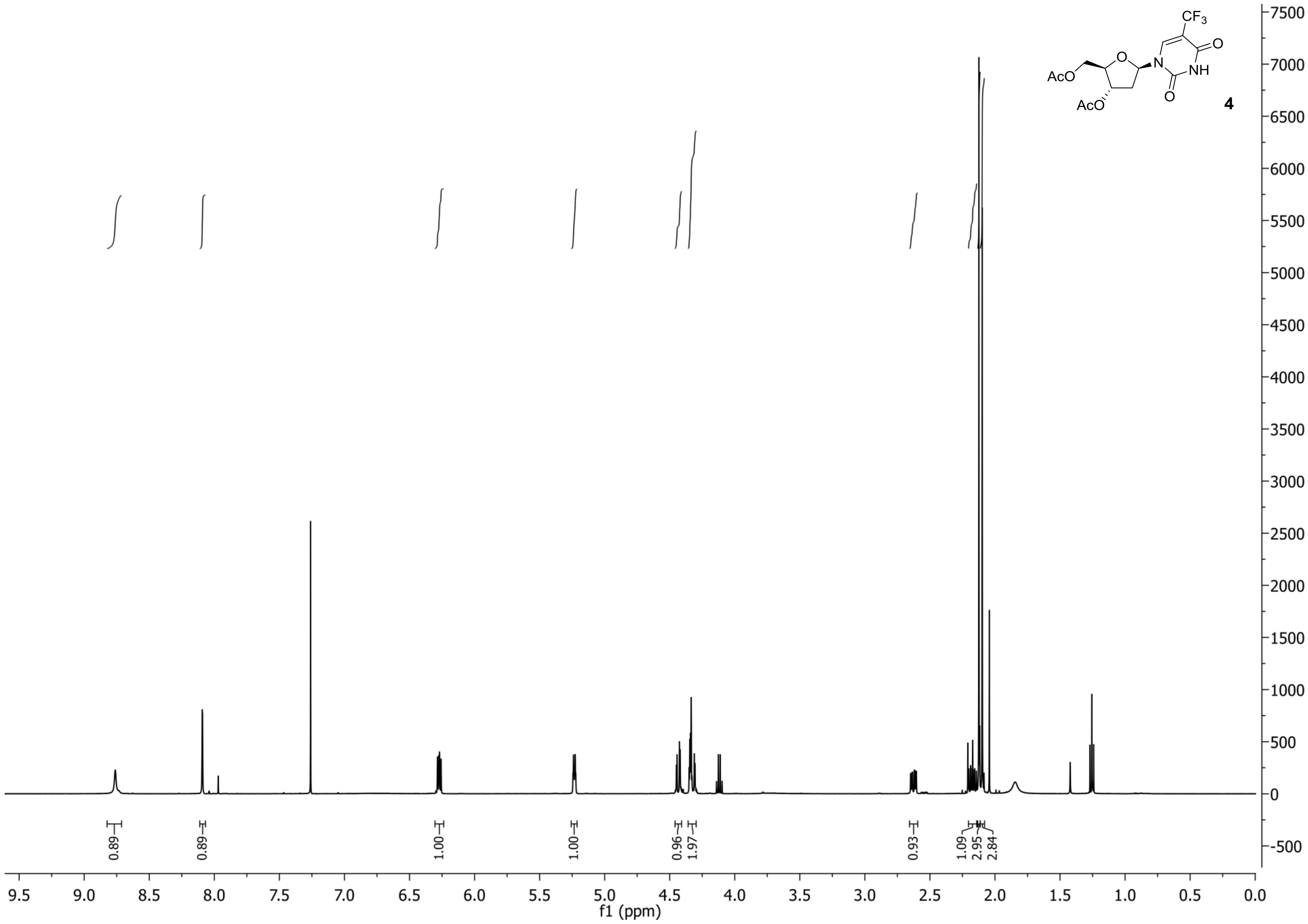

1.6. ^{13}C NMR compound **4**

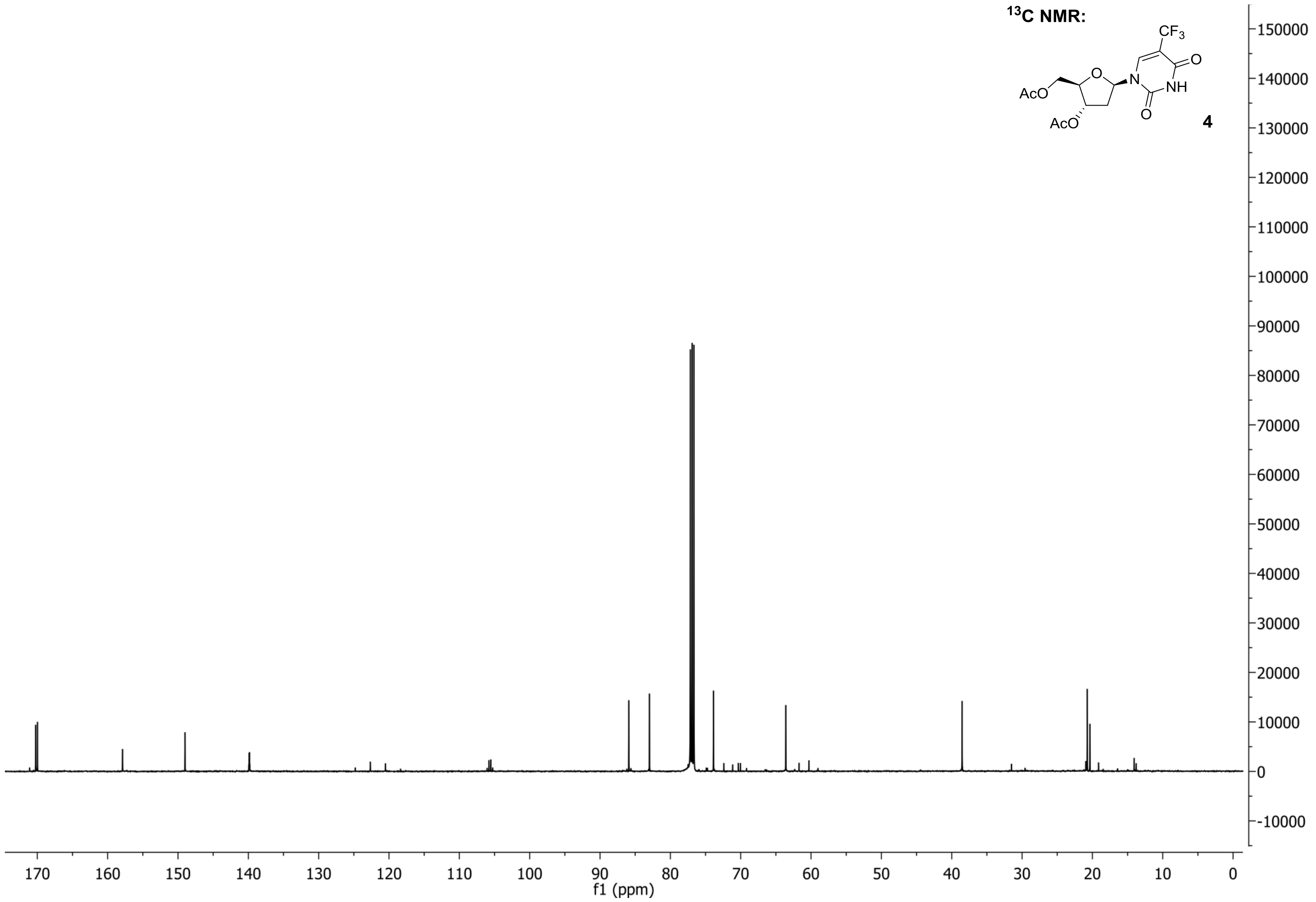

1.7. ^{1}H NMR **TFT**

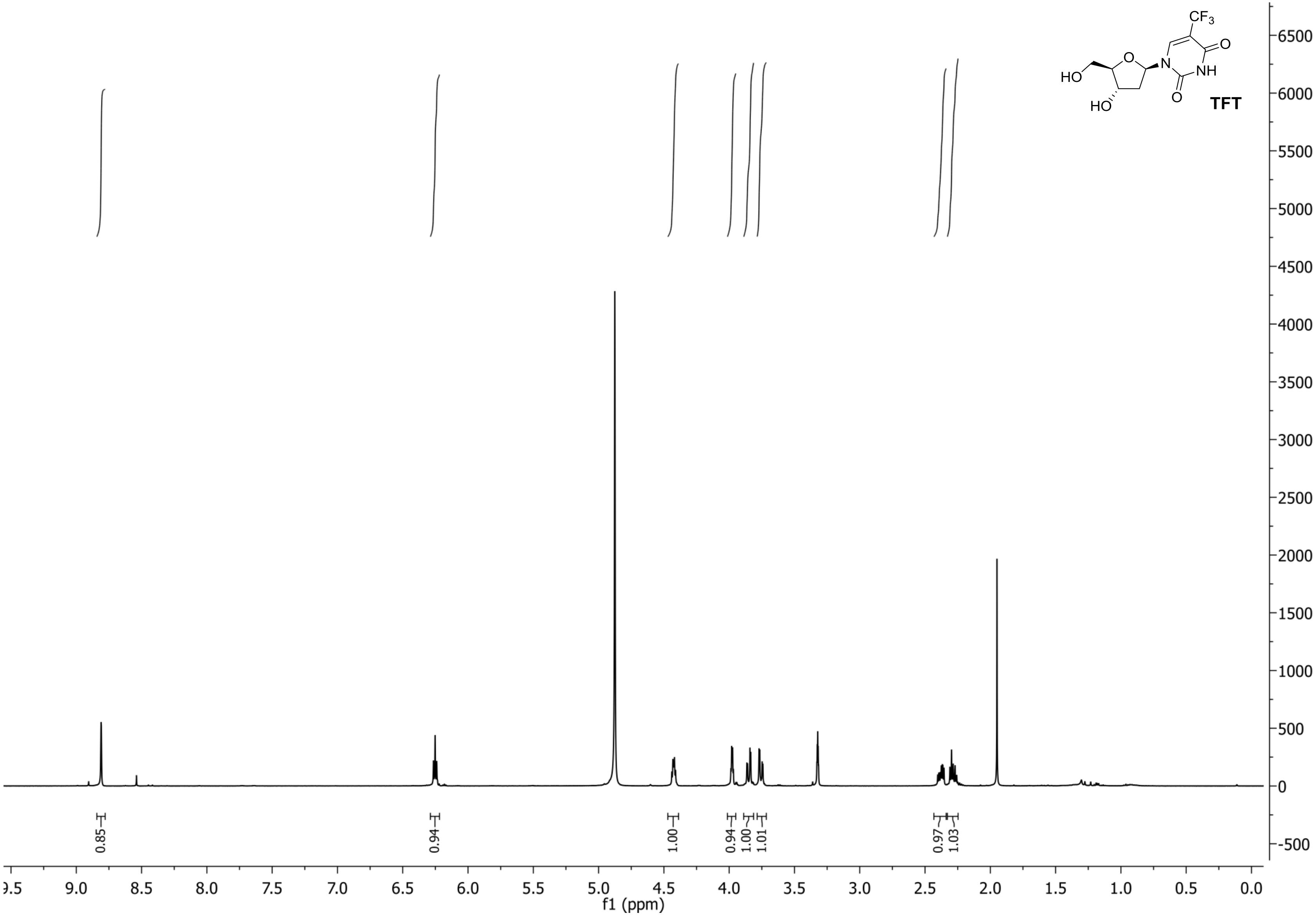
CF3
O
O
N
NH
HO
HO
O
TFT
0.85
0.94
1.00
0.94
1.00
1.01
0.97
1.03
9.5
9.0
8.5
8.0
7.5
7.0
6.5
6.0
5.5
5.0
4.5
4.0
3.5
3.0
2.5
2.0
1.5
1.0
0.5
0.0
f1 (ppm)
6500
6000
5500
5000
4500
4000
3500
3000
2500
2000
1500
1000
500
0
-500

1.8. ^{13}C NMR **TFT**

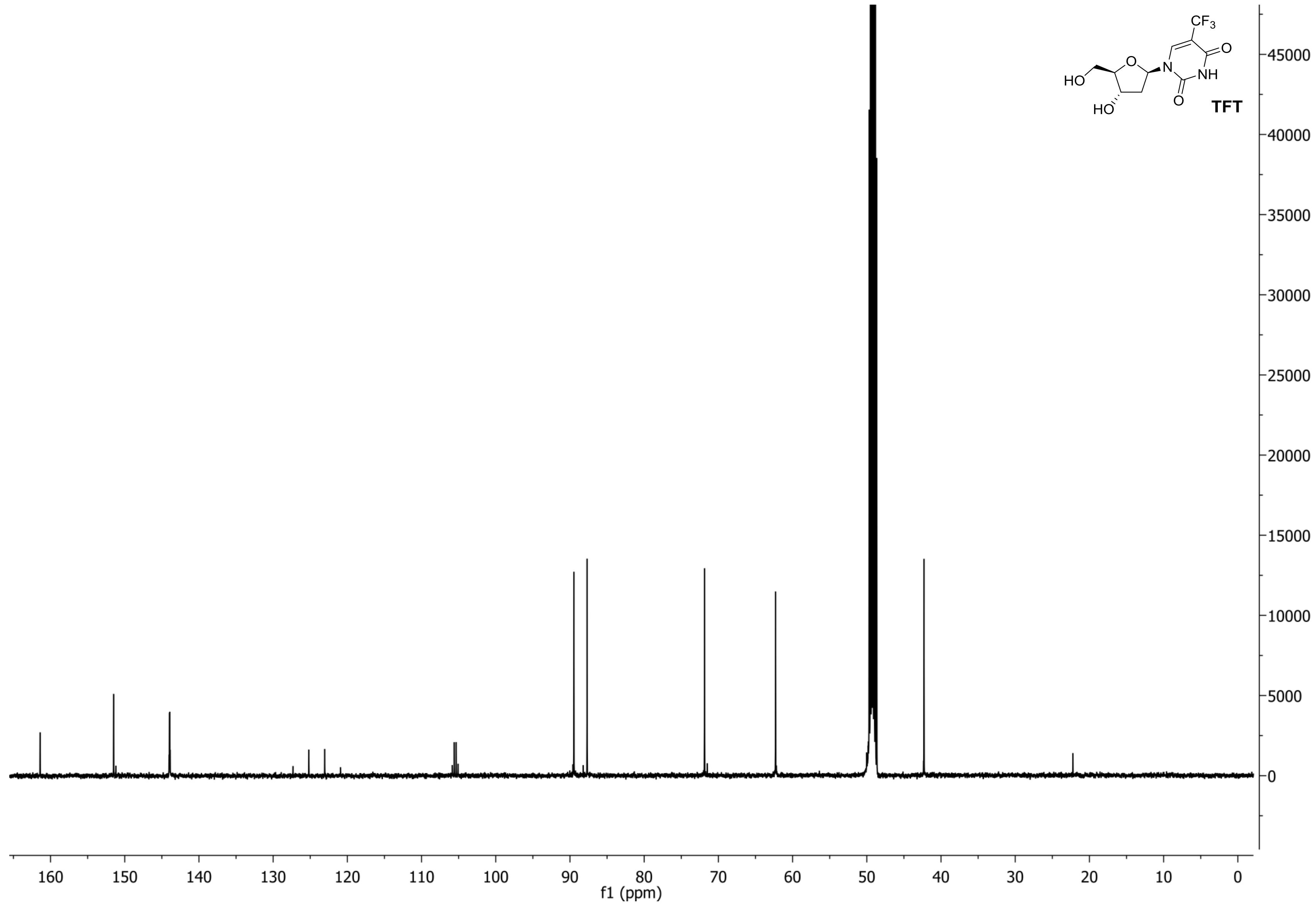

1.9. ^{1}H NMR **Mbh-Cl**

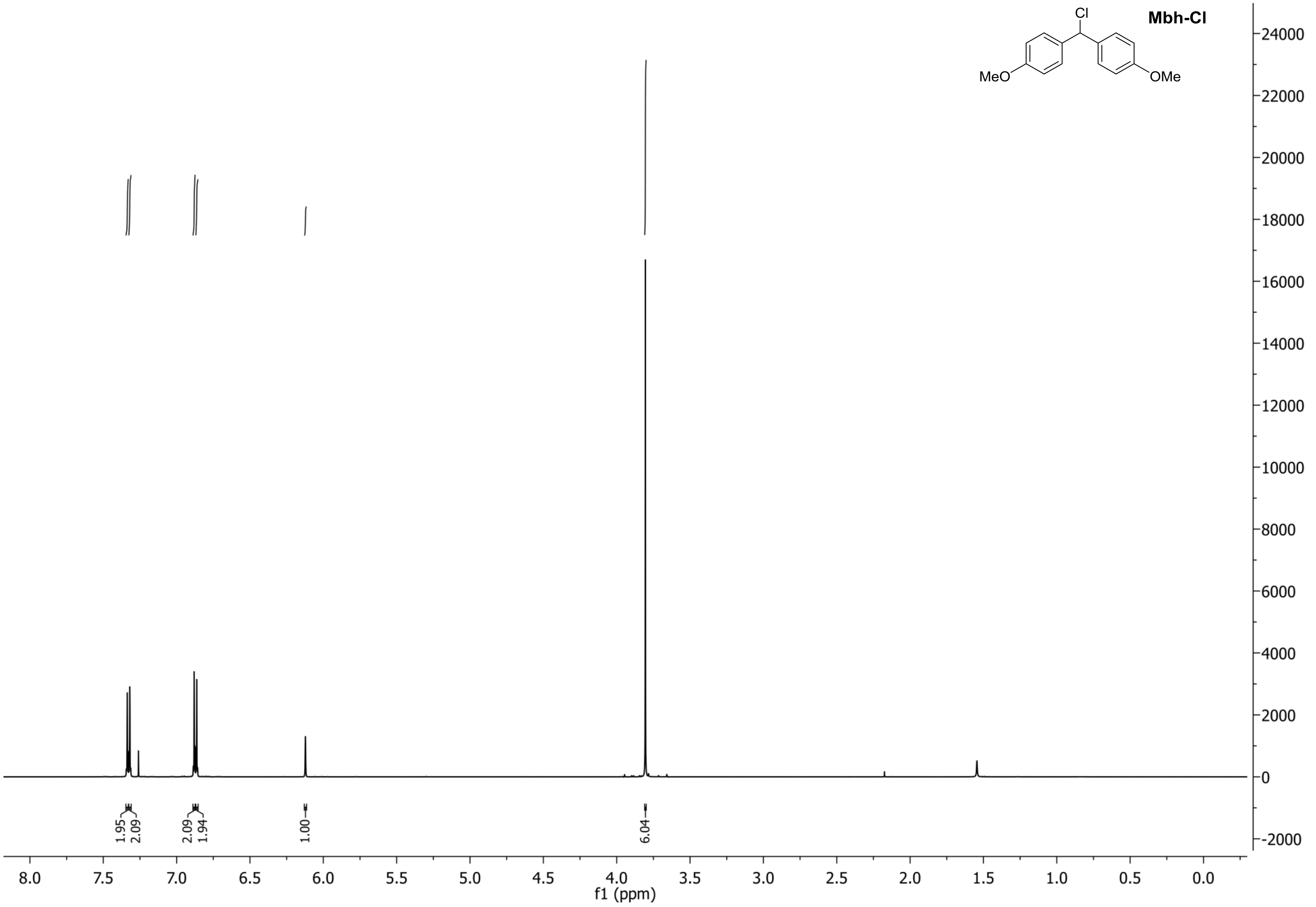

1.10. ^{13}C NMR **Mbh-Cl**

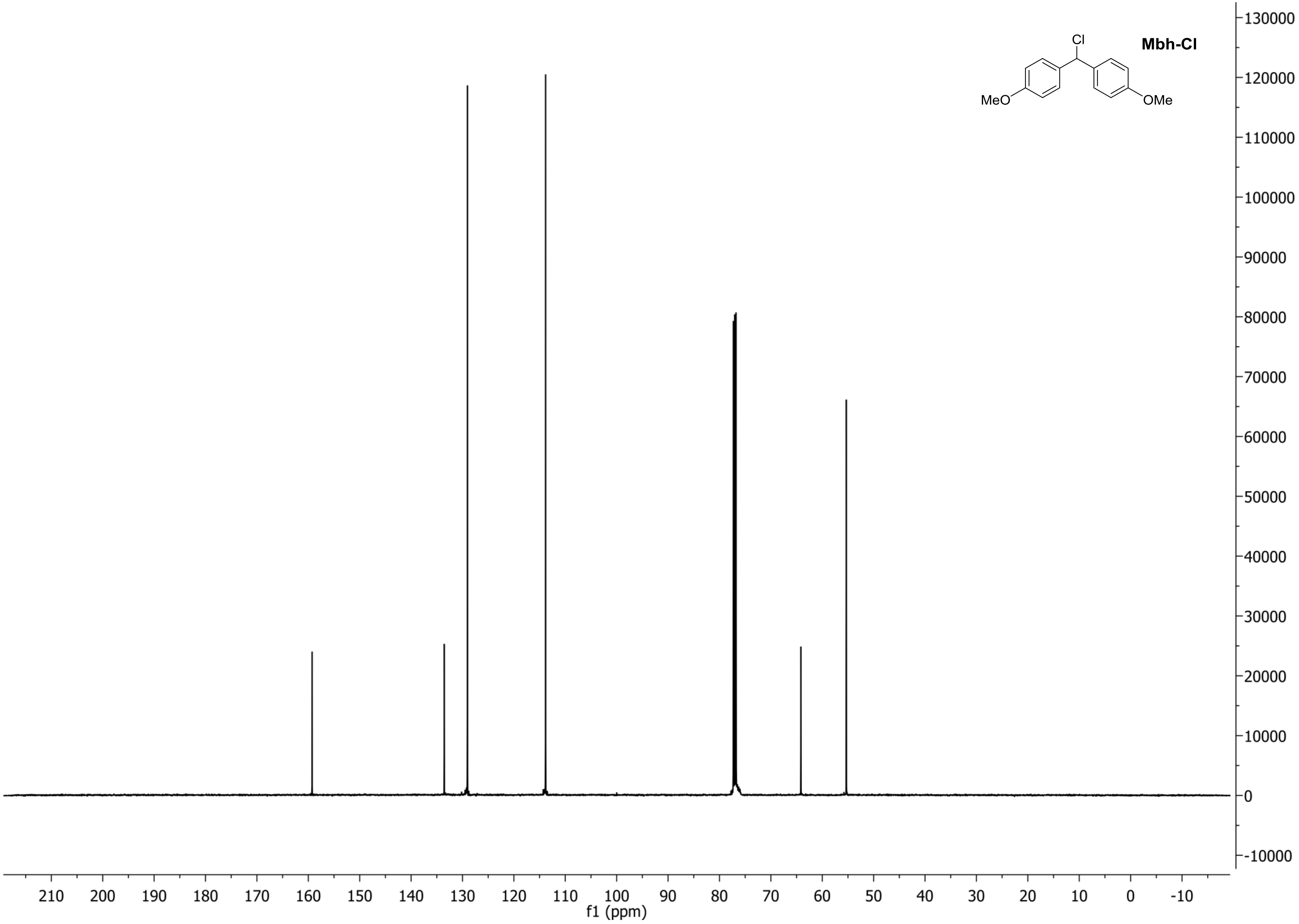

2. Radiochemistry

2.1. [18F]TFT automation

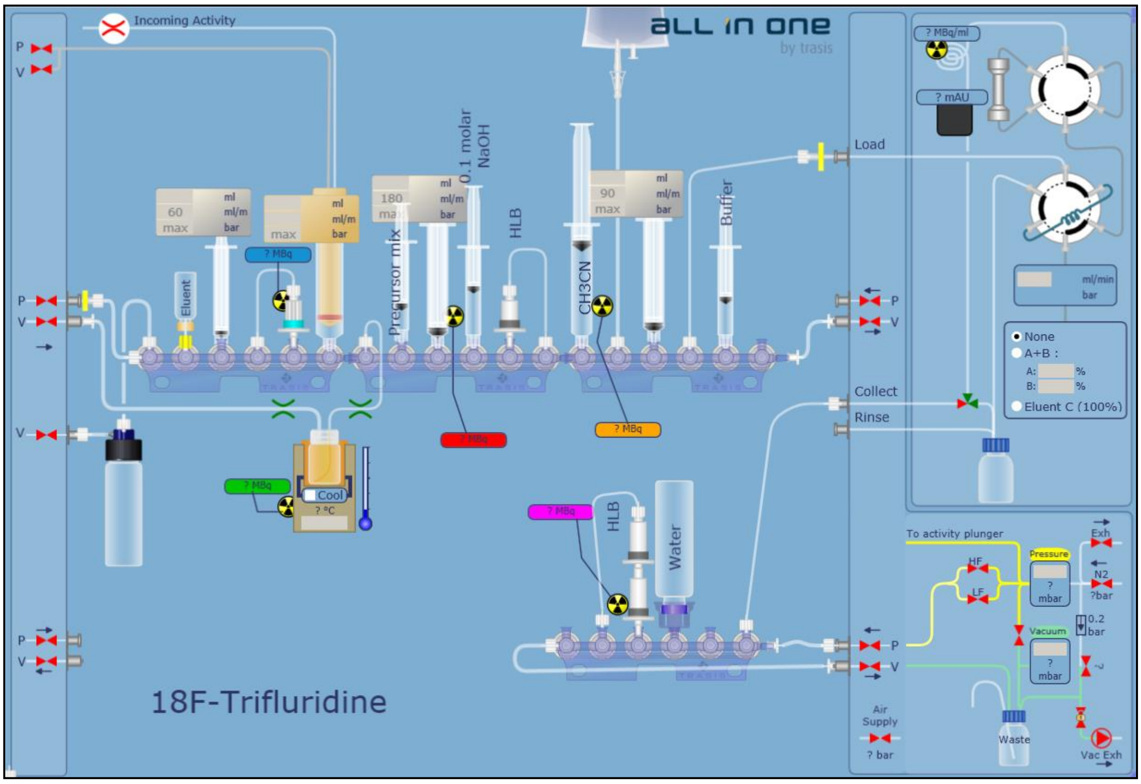

Figure S1. Trasis AllInOne automated synthesis cassette setup for the automatic preparation of [^{18}F]TFT.

2.2. Characterisation of **[^{18}F]-4** and [^{18}F]TFT by radio-HPLC

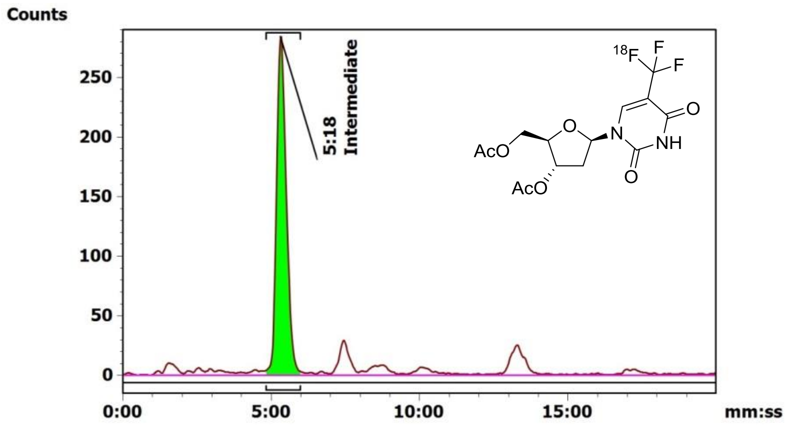

Figure S2. Radio-HPLC chromatogram of the radiolabelled intermediate **[^{18}F]-4** (retention time 5:18 minutes:seconds, purified using a Sep-pak SPE HLB cartridge only). Column: Luna® C18, 4.6 × 150 mm, 5 µM (Phenomenex, UK). Isocratic method 1: eluant A H_2O, 65%; eluant B CH_3CN, 35%; flow rate 1 mL/min.

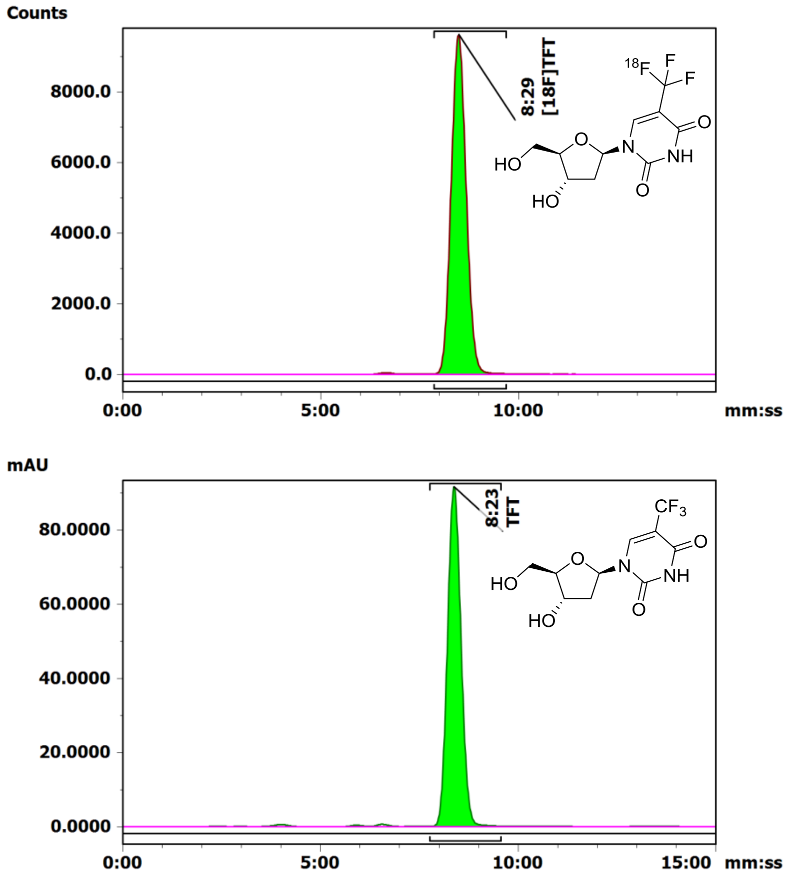

Figure S3. Radio-HPLC chromatogram of [^{18}F]TFT (retention time 8:29 minutes:seconds) and the corresponding UV profile, after semi-preparative HPLC purification (UV signal is detected earlier than the radio-signal due to the distance between the two detectors). Column: Luna® C18, 4.6 × 150 mm, 5 µM (Phenomenex, UK). Isocratic method 2: eluant A H_2O, 90%; eluant B CH_3CN, 10%; flow rate 1 mL/min.

2.3. Determination of [^{18}F]TFT molar activity

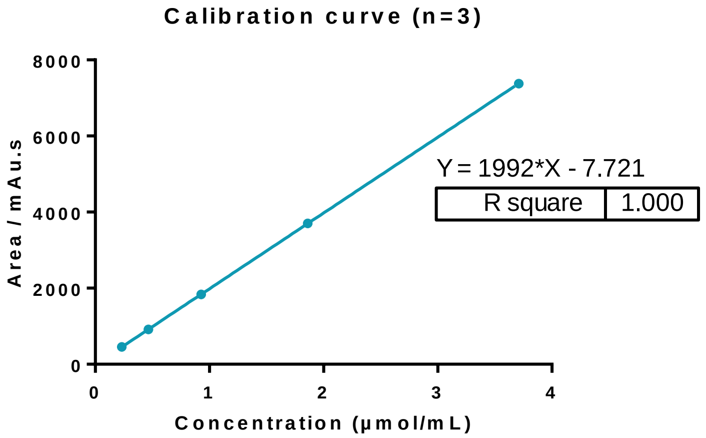

Figure S4. Calibration curve generated from [^{19}F]TFT standard for determination of molar activity. [^{19}F]TFT was injected onto the HPLC (5 µL injection volume) at 5 different concentrations (n=3) in H_2O, and the area under the curve was measured. By injecting the same volume of [^{18}F]TFT (X GBq/mL) and observing the area under the curve, the concentration of the sample can be ascertained (X µmol/mL), and hence the molar activity (X GBq/µmol). See Figure S3 for details of the HPLC method and column type.

2.4. [18F]FLT radiosynthesis procedure

[18F]FLT was synthesised on the Trasis AllInOne™ synthesis module using an [18F]FLT cassette (reference no. S4000-8842, Trasis Belgium) and reagent kit (product no. PEFL-0065-R, ABX, Germany). The radiosynthesis procedure was derived from a protocol supplied by Trasis. [18F]FLT was isolated in an overall RCY of 9% ± 1.03 (n=3), with a molar activity of 47.2 GBq/µmol ± 2.62 (n=3). For *in vivo* biodistribution experiments, the molar activity of [18F]FLT was lowered to 0.1 GBq/µmol (n=1) by adding an appropriate concentration of [19F]FLT (124 µg [19F]FLT was added to 24.9 MBq [18F]FLT in 370 µL saline).

3. LogD$_{7.4}$

[18F]TFT (~0.074 MBq in 2.6 µL) was added to PBS (pH 7.4, 0.5 mL) and n-octanol (0.5 mL). The mixture was vortexed for 10 minutes and centrifuged at 10,000 × *g* for 10 minutes. The experiment was performed in triplicate. Three 100 µl samples were taken from each layer and the amount of radioactivity in each aliquot was measured in a 2480 WIZARD^2 automatic gamma counter (Perkin Elmer, UK) as counts per minutes. The distribution coefficient at pH 7.4 (LogD$_{7.4}$) was expressed as the mean ± standard deviation, and was calculated using the formula:

$$LogD_{7.4} = \log[\frac{counts\ octanol}{counts\ PBS}]$$

[18F]TFT LogD$_{7.4}$ = – 0.56 ± 0.014

4. Radiotracer stability in H_2O at ambient temperature

[18F]TFT (8.05 MBq) in 200 µL H_2O was added to a vial at ambient temperature, without stirring. The sample was analysed by HPLC 0, 1, 3 and 5 hours post-reformulation to assess stability (i.e. defluorination, radiolysis). The peak with the retention time of 8:29 minutes:seconds was characterised as the product, and % intact radiotracer was determined from the area under this peak (% region of interest). A radiolabelled impurity (retention time 6:39 minutes:seconds) was observed immediately after reformulation (0 hours), and the area under the impurity grew slightly over time. By 5 hours, another small impurity (retention time 3:41 minutes:seconds) had formed, yet the % intact radiotracer remained as high as 99.2%. No defluorination was observed.

Radiotracer stability: (Intact radiotracer at 5 hours/Intact radiotracer at 0 hours)*100 = **99.7%**

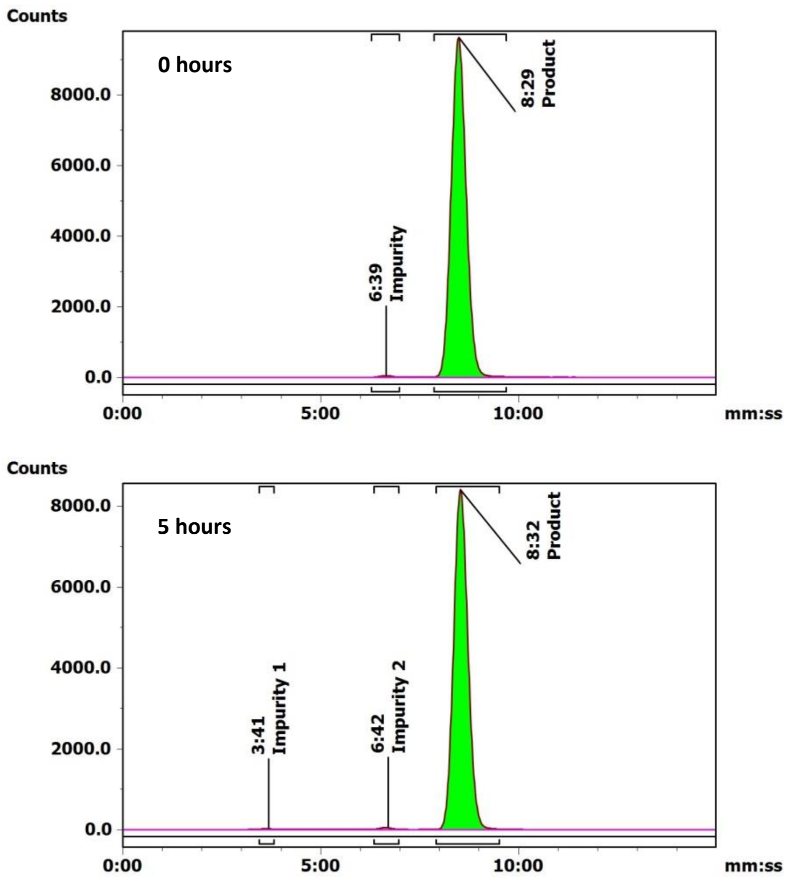

Figure S5. Radio-HPLC chromatograms representing [^{18}F]TFT stability after 0 and 5 hours in H_2O at ambient temperature. HPLC details: Luna® C18 column, 4.6 × 150 mm, 5 µM (Phenomenex, UK). Isocratic method 2*:* Eluant A H_2O, 90%; eluant B CH_3CN, 10%; flow rate 1 mL/min.

Table S1. % Intact radiotracer remaining after storage in H_2O at ambient temperature (no stirring). % Intact radiotracer was derived from the area under the [^{18}F]TFT product peak (retention time: 8:29 – 8:32 minutes:seconds) as a percentage of the total radiolabelled products.

**Intact radiotracer remaining %**			
**0 hours**	**1 hour**	**3 hours**	**5 hours**
99.5	99.5	99.4	99.2

5. *In vitro* thymidine phosphorylase assay

5.1. Procedure

As described in the literature,[1] to a solution of human recombinant thymidine phosphorylase (1 mg/mL, product no. ab101169, Abcam, UK) (1 µL) in 0.17 mM K_2HPO_4 (pH 7.6) (99 µL) was added a solution of radiotracer ([^{18}F]TFT or [^{18}F]FLT, 5 MBq) in a 0.17 mM K_2HPO_4 (pH 7.6)/ethanol mixture (9:1, 200 µL). The sample was incubated at 37 °C for 30 minutes. Trifluoroacetic acid (30 µL) was added, followed by addition of ice-cold methanol (600 µL). The mixture was centrifuged at 4 °C (12,000 × *g*) for 10 minutes, and the supernatant was removed and evaporated to dryness. The residue was reconstituted into a H_2O/acetonitrile mixture (9:1, 300 µL) and monitored by RP-HPLC. The experiments were performed in triplicate.

5.2. HPLC data

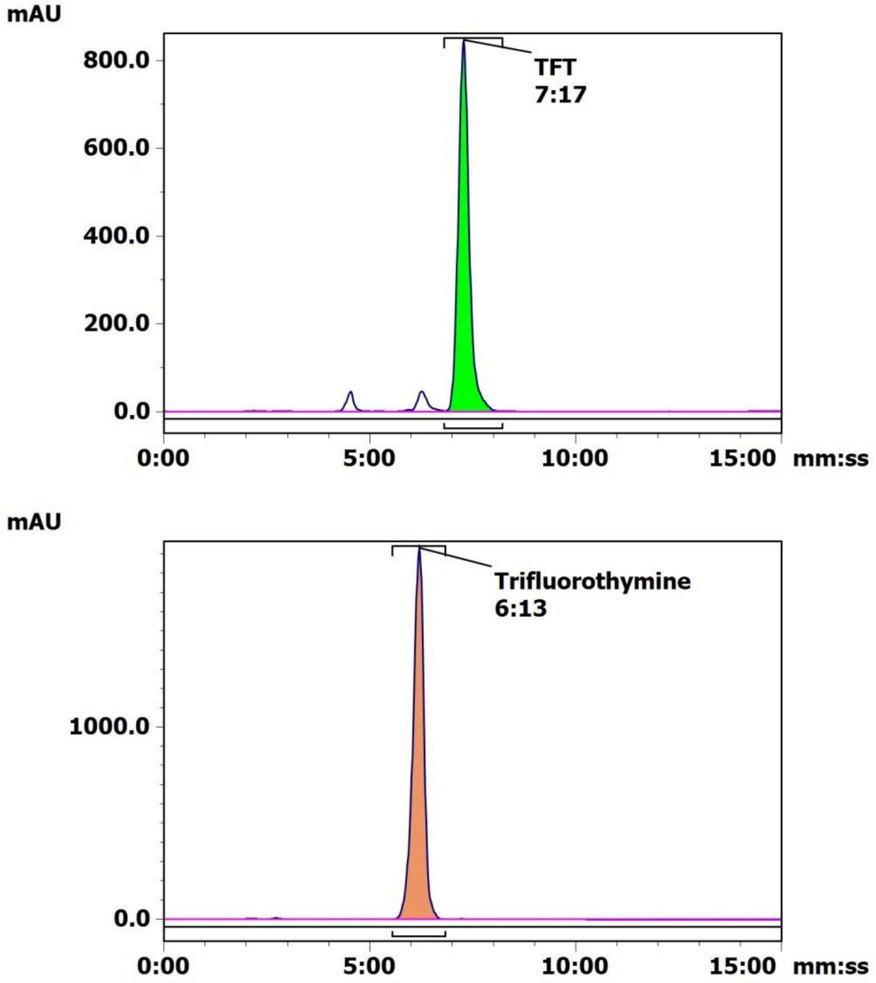

Figure S6. HPLC chromatograms showing the UV absorbance signals of the parent compound TFT **5** (retention time 7:17 minute:seconds) and the expected metabolite trifluorothymine (retention time 6:13 minute:seconds). HPLC details: µBondapak C18 column, 7.8 × 300 mm, 10 µm, 125 Å (Waters, UK) and isocratic method 4*:* eluant A H_2O, 90%; eluant B EtOH, 10%; 3 mL/min flow rate.

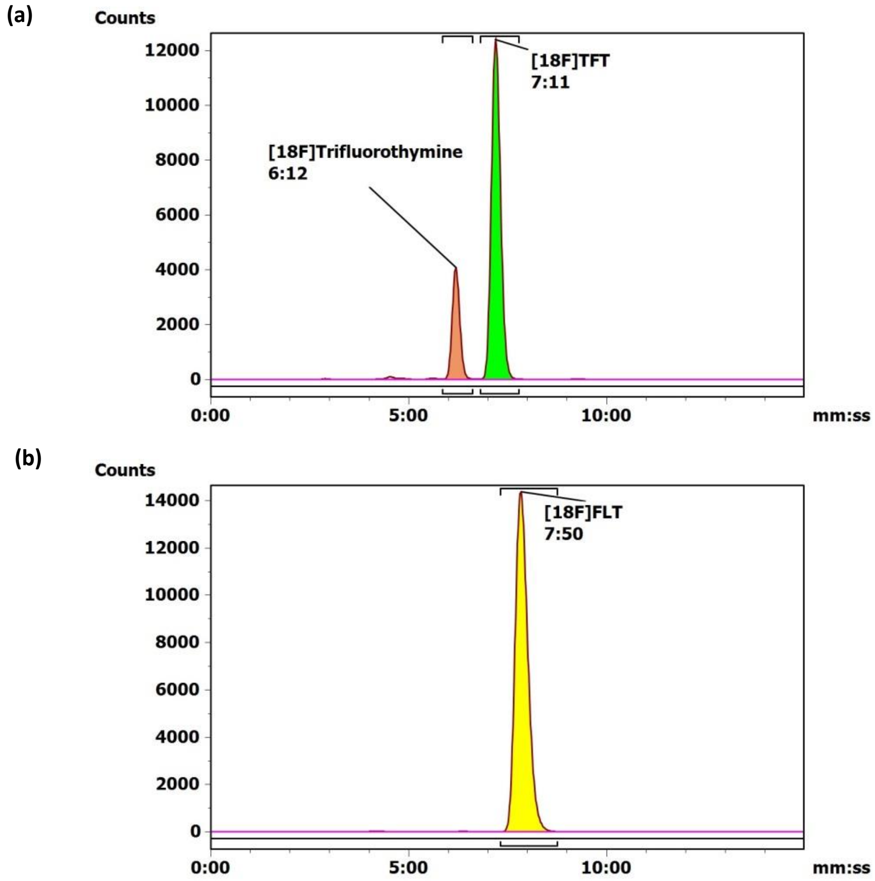

Figure S7. (a) Representative HPLC trace of [^{18}F]TFT after incubating with thymidine phosphorylase, showing the parent compound (retention time 7:11 minute:seconds) and the metabolite [^{18}F]trifluorothymine (retention time 6:12 minute:seconds); **(b)** Representative HPLC trace of [^{18}F]FLT after incubating with thymidine phosphorylase, showing the parent compound (retention time 7:50 minute:seconds) only. HPLC details: µBondapak C18 column, 7.8 × 300 mm, 10 µm, 125 Å (Waters, UK) and isocratic method 4*:* eluant A H_2O, 90%; eluant B EtOH, 10%; 3 mL/min flow rate.

5.3. Results

Table S2. Thymidine phosphorylase-mediate metabolism of [^{18}F]TFT and [^{18}F]FLT based on HPLC analysis. The data was quantified based on the area under the curve (region of interest) for [^{18}F]TFT (retention time 7:11 minute:seconds), [^{18}F]trifluorothymine (retention time 6:12 minute:seconds), and [^{18}F]FLT (retention time 7:50 minute:seconds), and expressed as a percentage.

	% Region of interest from HPLC chromatogram (Mean ± SD)	
	[^{18}F]TFT	[^{18}F]FLT
Parent compound	78.6 ± 0.0666	100 ± 0
Metabolite	21.4 ± 0.0666	0 ± 0

6. Cell homogenates assay

6.1. Procedure

Standard experiment: HCT116 cell homogenates were prepared as reported in the literature.[2] A reaction mixture was prepared containing the HCT116 cell homogenate (0.1 mg, 20 µL), radiotracer ([^{18}F]TFT or [^{18}F]FLT 0.5 – 0.8 MBq, 1 µL), 1 M Tris-HCl (pH 7.5) (5 µL), 0.25 M ATP (1 µL), 0.25 M $MgCl_2$ (1 µL), and deionised H_2O to a final volume of 100 µL. The mixture was incubated at 37 °C for 60 minutes. Samples were diluted with ice-cold H_2O/acetonitrile mixture (9:1, 1 mL), passed through a Millex 0.2 µm filter (Millipore, Billerica, MA, USA) and monitored by RP-HPLC. The experiments were performed in triplicate.

Control experiment 1 (dephosphorylation): A reaction mixture was prepared containing the HCT116 cell homogenate (0.1 mg, 20 µL), radiotracer ([^{18}F]TFT or [^{18}F]FLT 0.5 – 0.8 MBq, 1 µL), 1 M Tris-HCl (pH 7.5) (5 µL), 0.25 M ATP (1 µL), 0.25 M $MgCl_2$ (1 µL), and deionised H_2O to a final volume of 100 µL. The mixture was incubated at 37 °C for 60 minutes. Subsequently, bacterial alkaline phosphatase (5 enzyme units/µL, product no. 18011015, ThermoFisher Scientific) (45 µL) was and the mixture was incubated at 37 °C for a further 60 minutes. The samples were processed as previously.

Control experiment 2 (no ATP): A reaction mixture was prepared containing the HCT116 cell homogenate (0.1 mg, 20 µL), radiotracer ([^{18}F]TFT or [^{18}F]FLT 0.5 – 0.8 MBq, 1 µL), 1 M Tris-HCl (pH 7.5) (5 µL), 0.25 M $MgCl_2$ (1 µL), and deionised H_2O to a final volume of 100 µL. The mixture was incubated at 37 °C for 60 minutes. The samples were processed as previously.

6.2. HPLC data

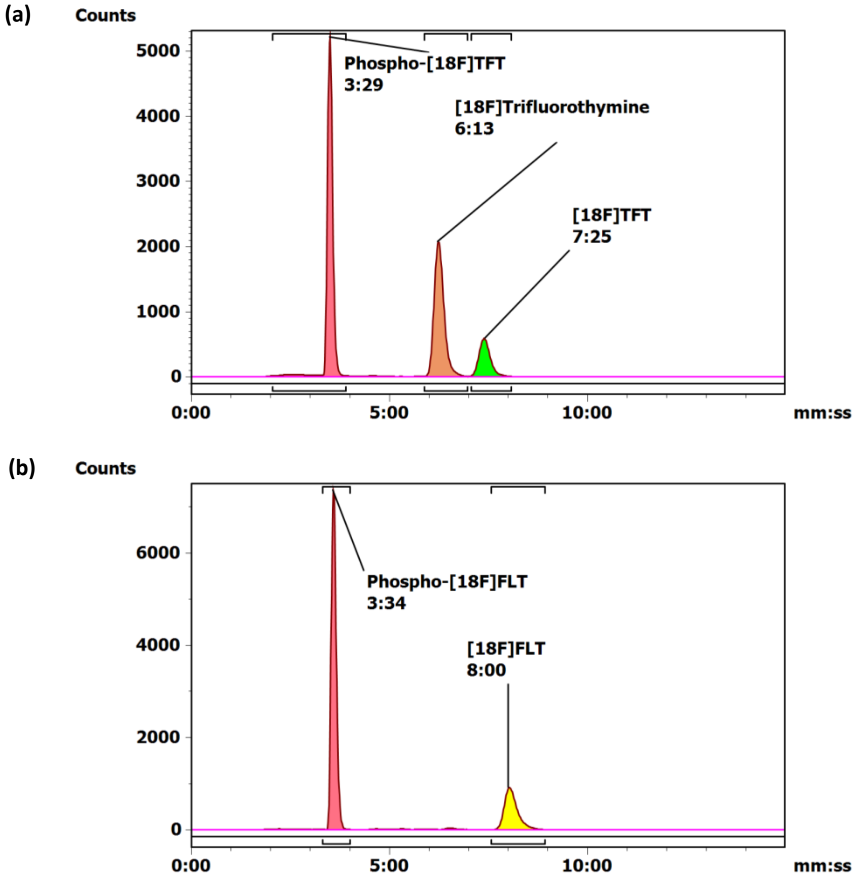

Figure S8. (a) Representative HPLC trace of [^{18}F]TFT after incubation with cell homogenates and ATP for 60 minutes. Parent [^{18}F]TFT (retention time 7:25 minutes:seconds), [^{18}F]trifluorothymine (retention time 6:13 minutes:seconds) and the phosphorylated adduct of [^{18}F]TFT (retention time 3:29 minutes:seconds) were observed. **(b)** Representative HPLC trace of [^{18}F]FLT after incubation with cell homogenates and ATP for 60 minutes. Parent [^{18}F]FLT (retention time 8:00 minutes:seconds) and the phosphorylated adduct of [^{18}F]FLT (retention time 3:34 minutes:seconds) were observed. HPLC details: µBondapak C18 column, 7.8 × 300 mm, 10 µm, 125 Å (Waters, UK) and isocratic method 4*:* eluant A H_2O, 90%; eluant B EtOH, 10%; 3 mL/min flow rate.

6.3. Results

Table S3. Metabolism of [^{18}F]TFT after incubation with HCT116 cell homogenates based on HPLC analysis. The data was quantified based on the area under the curve (region of interest) for [^{18}F]TFT (retention time 7:25 minute:seconds), [^{18}F]trifluorothymine (retention time 6:13 minute:seconds), and the phosphorylated adduct of [^{18}F]TFT (retention time 3:29 minute:seconds), and expressed as a percentage (mean ± SD). Standard experiment 1 was compared with control experiment 1 and control experiment 2. In control experiment 1, a dephosphorylation enzyme was included after initial incubation, to demonstrate that the product with retention time 3:29 minutes:seconds was a phosphorylated adduct. Conversion of phospho-[^{18}F]TFT back to the parent compound was observed. In control experiment 2, no ATP was added, in order to limit the formation of phospho-[^{18}F]TFT. The presence of phospho-[^{18}F]TFT was largely reduced.

	Standard experiment 1 (60 minute incubation)	Control experiment 1 (dephosphorylation)	Control experiment 2 (no ATP)
Parent [^{18}F]TFT	16.7 ± 6.61	30.6 ± 1.96	69.0 ± 2.05
[^{18}F]Trifluorothymine	37.8 ± 0.947	47.5 ± 1.37	27.7 ± 1.07
Phospho-[^{18}F]TFT	45.4 ± 7.47	21.9 ± 2.51	3.32 ± 0.997

Table S4. Metabolism of [^{18}F]FLT after incubation with HCT116 cell homogenates based on HPLC analysis. The data was quantified based on the area under the curve (region of interest) for [^{18}F]FLT (retention time 8:00 minute:seconds) and the phosphorylated adduct of [^{18}F]FLT (retention time 3:34 minute:seconds), and expressed as a percentage (mean ± SD). In control experiment 1, conversion of phospho-[^{18}F]FLT back to the parent compound was observed. In control experiment 2, the presence of phospho-[^{18}F]FLT was largely reduced.

	Standard experiment 1 (60 minute incubation)	Control experiment 1 (dephosphorylation)	Control experiment 2 (no ATP)
Parent [^{18}F]FLT	22.3 ± 1.20	63.0 ± 3.68	96.1 ± 0
Phospho-[^{18}F]FLT	77.7. ± 1.20	37.0 ± 3.68	3.90 ± 0

7. In vivo metabolite analysis

7.1. HPLC data

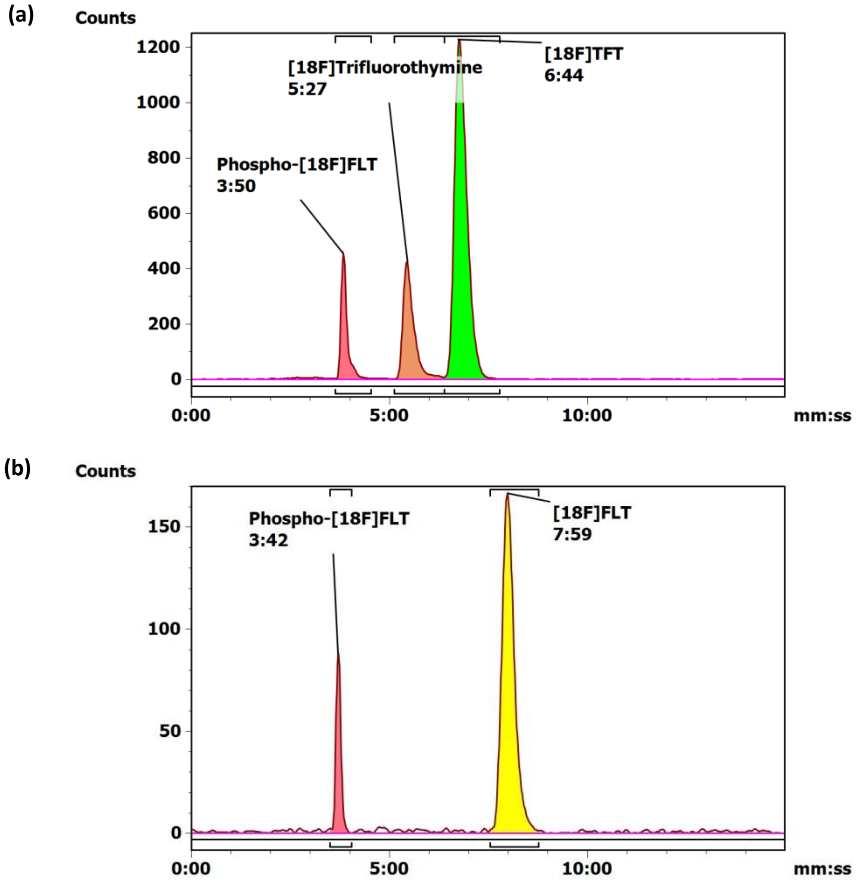

Figure S9. (a) Representative HPLC trace from *in vivo* metabolite analysis of [^{18}F]TFT (taken from a plasma sample). Parent [^{18}F]TFT (retention time 6:44 minutes:seconds), [^{18}F]trifluorothymine (retention time 5:27 minutes:seconds) and the phosphorylated adduct of [^{18}F]TFT (retention time 3:50 minutes:seconds) were observed across all liver, tumour and plasma samples. **(b)** Representative HPLC trace from *in vivo* metabolite analysis of [^{18}F]FLT (taken from a tumour sample). Parent [^{18}F]FLT (retention time 7:59 minutes:seconds) and the phosphorylated adduct of [^{18}F]FLT (retention time 3:42 minutes:seconds) were observed across all liver, tumour and plasma samples.. HPLC details: µBondapak C18 column, 7.8 × 300 mm, 10 µm, 125 Å (Waters, UK) and isocratic method 4*:* eluant A H_2O, 90%; eluant B EtOH, 10%; 3 mL/min flow rate.

7.2. Results

Table S5. *In vivo* metabolism of [^{18}F]TFT based on HPLC analysis. The data was quantified based on the area under the curve (region of interest) for [^{18}F]TFT (retention time 6:44 minute:seconds), [^{18}F]trifluorothymine (retention time 5:27 minute:seconds), and the phosphorylated adduct of [^{18}F]TFT (retention time 3:50 minute:seconds), and expressed as a percentage (mean ± SD). Liver, tumour, and plasma samples were analysed, with mouse sacrifice and sample acquisition at two different time points post-injection of [^{18}F]TFT.

	Liver		Tumour		Plasma	
	15 min	60 min	15 min	60 min	15 min	60 min
Parent [^{18}F]TFT	2.33 ± 1.19	0.75 ± 0.435	15.9 ± 6.83	8.36 ± 6.16	63.2 ± 5.70	38.3 ± 6.96
[^{18}F]Trifluoro-thymine	1.58 ± 0.528	1.47 ± 1.33	75.0 ± 2.89	49.8 ± 8.78	16.6 ± 4.71	24.7 ± 2.37
Phospho-[^{18}F]TFT	96.1 ± 1.70	97.8 ± 1.76	9.08 ± 4.34	41.9 ± 14.7	20.1 ± 10.4	37.1 ± 6.07

Table S6. *In vivo* metabolism of [^{18}F]FLT based on HPLC analysis. The data was quantified based on the area under the curve (region of interest) for [^{18}F]FLT (retention time 7:59 minute:seconds) and the phosphorylated adduct of [^{18}F]FLT (retention time 3:42 minute:seconds), and expressed as a percentage (mean ± SD). Liver, tumour, and plasma samples were analysed, with mouse sacrifice and sample acquisition at two different time points post-injection of [^{18}F]FLT.

	Liver		Tumour		Plasma	
	15 min	60 min	15 min	60 min	15 min	60 min
Parent [^{18}F]TFT	94.4 ± 0.751	92.2 ± 2.17	87.2 ± 7.72	77.7 ± 15.3	98.1 ± 0.580	96.4 ± 0.745
Phospho-[^{18}F]TFT	5.58 ± 0.751	7.82 ± 2.17	12.8 ± 7.72	22.3 ± 15.3	1.86 ± 0.580	3.65 ± 0.745

8. Biodistribution studies

Table S7. Biodistribution results gathered using HCT116 tumour-bearing mice, following administration of [^{18}F]TFT (~10 MBq per mouse, 0.4 GBq/µmol) at 15 and 60 minutes post-injection, and of [^{18}F]FLT (~10 MBq per mouse, 0.1 GBq/µmol) at 60 minutes post-injection. Each experiment was performed in triplicate and the data are reported as the %ID/g ± SD.

	**% ID/g ± SD**		
**Organ**	**[^{18}F]TFT 15 min**	**[^{18}F]TFT 60 min**	**[^{18}F]FLT 60 min**
Blood	7.94 ± 1.21	1.84 ± 0.186	2.64 ± 0.260
Heart	2.83 ± 0.324	0.722 ± 0.0703	2.23 ± 0.167
Lung	4.11 ± 0.531	1.34 ± 0.255	2.13 ± 0.210
Kidney	14.7 ± 3.21	6.15 ± 0.339	4.09 ± 0.180
Spleen	3.69 ± 0.215	2.51 ± 0.305	2.72 ± 0.295
Liver	13.8 ± 2.44	6.13 ± 1.25	2.69 ± 0.121
Pancreas	2.05 ± 0.264	0.700 ± 0.237	2.06 ± 0.290
Bone	2.72 ± 0.485	1.77 ± 1.33	1.68 ± 0.134
Stomach	1.79 ± 0.243	0.632 ± 0.116	1.62 ± 0.200
Small intestine	3.87 ± 0.403	3.19 ± 0.238	3.34 ± 0.216
Large intestine	3.91 ± 0.961	2.26 ± 0.178	2.89 ± 0.411
Muscle	1.23 ± 0.0547	0.689 ± 0.595	1.98 ± 0.215
Tumour	3.67 ± 1.28	2.49 ± 0.0742	8.32 ± 1.28

Table S8. Tumour/organ ratios for [18F]TFT 15 and 60 minutes post-injection, and for [18F]FLT 60 minutes post-injection. The ratios were calculated using the following formula: (Tumour %ID/g) / (Organ %ID/g).

Organ	(Tumour %ID/g) / (Organ %ID/g)		
	[18F]TFT 15 min	[18F]TFT 60 min	[18F]FLT 60 min
Blood	0.474 ± 0.183	1.36 ± 0.145	3.19 ± 0.741
Heart	1.33 ± 0.522	3.46 ± 0.237	3.77 ± 0.819
Lung	0.905 ± 0.323	1.91 ± 0.330	3.96 ± 0.931
Kidney	0.253 ± 0.080	0.406 ± 0.035	2.04 ± 0.341
Spleen	0.987 ± 0.312	1.00 ± 0.104	3.11 ± 0.766
Liver	0.268 ± 0.086	0.418 ± 0.084	3.11 ± 0.594
Pancreas	1.84 ± 0.749	3.79 ± 1.03	4.13 ± 1.13
Bone	1.34 ± 0.358	4.28 ± 5.74	5.02 ± 1.11
Stomach	2.06 ± 0.631	4.02 ± 0.700	5.24 ± 1.33
Small intestine	0.943 ± 0.284	0.784 ± 0.084	2.49 ± 0.272
Large intestine	0.986 ± 0.404	1.10 ± 0.057	2.92 ± 0.639
Muscle	3.00 ± 1.11	5.39 ± 3.13	4.25 ± 0.992

9. Dynamic PET scanning

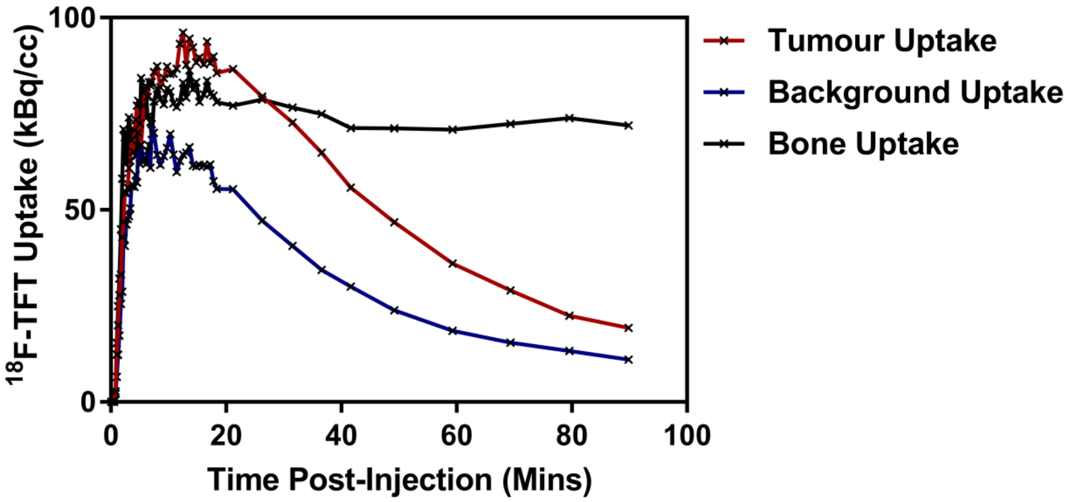

Figure S10. Quantitative results of the image analysis. [18F]TFT dynamic PET scans (0-90 minutes) were recorded and three volumes of interest were drawn around the tumour, the patella (to reflect the highest level of bone uptake), and the muscle (to reflect the background), using a 50% threshold. The mean counts were recorded and subsequently converted into kBq/cc. Beyond 20 minutes post-injection, tumour uptake decreased, while bone uptake was observed at a consistently high level.

References

1. B. D. Zlatopolskiy, A. Morgenroth, F. H.-G. Kunkel, E. A. Urusova, C. Dinger, T. Kull, C. Lepping and S. N. Reske, *J. Nucl. Med.*, 2009, **50**, 1895-1903.
2. G. Smith, R. Sala, L. Carroll, K. Behan, M. Glaser, E. Robins, Q.-D. Nguyen and E. O. Aboagye, *Nucl. Med. Biol.*, 2012, **39**, 652-665.
